# Incorporating transcriptomic data into genomic prediction models to improve the prediction accuracy of phenotypes of efficiency traits

**DOI:** 10.1186/s12711-025-01008-7

**Published:** 2025-10-23

**Authors:** Valentin P. Haas, Robin Wellmann, Pascal Duenk, Michael Oster, Siriluck Ponsuksili, Jörn Bennewitz, Mario P. L. Calus

**Affiliations:** 1 Animal Breeding and Genomics, Wageningen University & Research, Droevendaalsesteeg 1, 6700 AH Wageningen, The Netherlands; 2 Institute of Animal Science, University of Hohenheim, 70599 Stuttgart, Germany; 3 Research Institute for Farm Animal Biology (FBN), 18196 Dummerstorf, Germany

## Abstract

**Background:**

Since genomic selection has been established in animal breeding, attention has turned towards other omics layers that are seen as promising to improve prediction accuracy. Transcriptomic data provide insights into gene expression patterns, which are shaped by both genetic and environmental factors, offering a more comprehensive understanding of the expression of phenotypes. This study utilized various statistical methods to assess the applicability of transcriptomic data derived from intestinal tissue to the prediction of efficiency-related phenotypes. The focus was on formal derivation of the previously described GTCBLUP model, which was adapted to create GTCBLUPi and compared with other BLUP models. The GTCBLUPi model addresses redundant information between genomic and transcriptomic information. We compared estimated variance components and accuracies of prediction of phenotypes for efficiency-related traits in an F2 cross of 480 Japanese quail using different models. Additionally, we estimated transcriptomic correlations between the traits using animal effects based on transcriptomic similarity, and the effects of individual transcript abundances on the phenotypes.

**Results:**

This study showed that transcript abundances from the ileum explain a larger portion of the phenotypic variance of the traits than host genetics. Models incorporating both genetic and transcriptomic information outperformed those using only one type of information, with regard to the phenotypic variances explained. The combination of both data types resulted in higher trait prediction accuracies, confirming that transcriptomic information complements genetic data effectively. The derived GTCBLUPi model proved to be a suitable framework for integrating both information types. Additionally, polygenic backgrounds were identified for the traits studied based on transcriptomic profiles, along with high transcriptomic correlations between the traits.

**Conclusions:**

Transcriptomic data account for a high portion of phenotypic expression for all phenotypes and incorporating them enables more accurate predictions of phenotypes for efficiency and performance traits. Models that integrate both genetic and transcriptomic information are the most effective, offering valuable insights for improving phenotype prediction accuracy and insights in biological mechanisms underlying phenotypic variation of traits.

**Supplementary Information:**

The online version contains supplementary material available at 10.1186/s12711-025-01008-7.

## Background

Genomic selection refers to the use of genome-wide and dense single nucleotide polymorphisms (SNPs) to predict breeding values for phenotypic traits and the subsequent selection of individuals based on these estimated breeding values [1]. This approach has successfully improved the accuracy of breeding value estimation, especially for young individuals and has thereby accelerated genetic gain by reducing the generation interval [2, 3].

Generating multiple types of additional molecular data for larger numbers of individuals has become affordable in recent years. These highly informative, animal-specific data can provide valuable new layers of information for prediction of breeding values and phenotypes of traits, helping to further decipher the link between genomics and phenomics [4].

In addition to the widely used composition of gut microbiota (e.g. [5–7]), transcriptomic data are also promising predictors of phenotype, as they capture information on intermediates between DNA and the phenotypic trait [8]. Some studies have integrated transcriptomic data into genomic prediction models to improve prediction accuracies (e.g. [9–11]). When using intermediate data as an additional source of information in genomic best linear unbiased prediction (GBLUP) models, the challenge lies in the overlapping nature of the data layers. For example, the microbial composition in the gastrointestinal tract of animals is partially explained by host genetics (e.g. [12–14]) and the heritability of gene transcripts is in general high (e.g. [15–18]). Thus, using both SNP genotypes and other omics data as independent random effects in a mixed linear model leads to collinearity problems. Christensen et al. [19] and Perez et al. [11] proposed methods to address the redundancy between two different data layers in BLUP models. The Christensen et al. [19] method employs a two-step procedure that first estimates the total effect of omics data on phenotypes and then explicitly models the genetic portion of these omics effects in a second step. The extended BLUP model of Perez et al. [11] models genotype data and omics data conditioned on the genotypes simultaneously in a one-step approach, such that the modeled omics effects are purely non-genetic.

In the present study, we followed the idea of Perez et al. [11] and applied several models to a dataset consisting of 480 Japanese quails with genotypes, ileum tissue transcript abundances, and efficiency-related phenotypes [17, 20, 21]. Due to the standardized design of this experiment, this dataset is well-suited for providing deeper insight into the relationships between genomics, transcriptomics, and phenotypic traits, which can help identify suitable approaches for incorporating additional omics layers into genomic prediction.

The aim of this study was to evaluate and compare different mixed linear models that incorporate genomic and transcriptomic data. Specifically, we aimed to: (i) estimate the proportion of phenotypic variance explained by transcripts and genomic markers; (ii) evaluate the predictive performance of phenotypes of several BLUP models; (iii) estimate transcriptomic correlations between traits using animal effects based on transcriptomic similarity, and (iv) estimate the effect of individual transcript abundances on efficiency phenotypes to deepen our understanding of the relationships underlying phenotypes for efficiency traits. Particular emphasis was placed on deriving and applying a BLUP model that integrates both genomic and transcriptomic information while accounting for redundancy between these two data sources.

## Methods

### Experimental design and data collection

The dataset used in this study was collected at the University of Hohenheim (Germany). The experimental population comprises 480 F2 cross Japanese quail (*Coturnix japonica*) selected from an initial total of 920 animals, which were raised under controlled conditions and served as model animals for poultry species. The F2 generation was created by mating 12 males and 12 females from each founder line to produce the F1 generation. From the resulting F1 animals, 17 roosters and 34 hens were randomly selected and mated in a 1:2 ratio (one male with two females), resulting in a total of 920 F2 individuals. During the strong growing phase between the 10th and 15th day of life, the birds were allocated to metabolism units and fed an ad libitum corn-soybean meal-based diet. To let the birds express their full genetic potential of phosphorus utilization (PU), the feed contained a marginal P concentration without mineral P or phytase supplementation.

We used the following phenotypic traits: PU based on total P intake and P excretion in %, body weight gain (BWG) between days 10 and 15 in g, feed intake (FI) during this 5-day period in g, feed conversion ratio (FCR) as FI divided by the BWG in g/g, total amount of tibia ash in mg (TA), and calcium utilization (CaU) based on total Ca intake and Ca excretion in %. All quail were slaughtered on day 15, where blood and ileum mucosa samples samples were taken. Hatching and slaughter took place on 11 different test days, with a balanced animal number per test day and a balanced sex ratio. Details of the experimental design and phenotyping can be found in Beck et al. [20].

The animals were genotyped using a 6k Illumina iSelect chip, which resulted in 4k SNPs after filtering, for which a genetic linkage map was established [21]. For ileal microRNA (miRNA) and messenger RNA (mRNA) sequencing, discordant sib pairs were selected from each of ten families, with one sib exhibiting high and the other low PU [22, 23]. Same-sex siblings were selected and sex was balanced across families. Downstream analyses identified the top differentially expressed transcripts related to PU, including 77 miRNAs and 80 mRNAs [17]. Subsequently, the retrieved miRNA and mRNA candidates were assessed with 96.96 dynamic arrays on a Fluidigm BioMark HD system (Fluidigm Corporation, CA, USA) using a randomly selected subpopulation of 482 quails [17].

Estimates of heritabilities of the traits and of genetic and phenotypic correlations between the performance traits based on the full set of 920 animals have already been published [20, 24]. The data used here is a subset of these animals, as transcriptomic information was not available for all animals.

### Statistical analyses

All analyses using mixed linear models were executed using ASReml R (Version 4.1) [25] in R Studio (Version 4.2.3) [26]. Since the phenotypes showed a highly skewed distribution, we applied a Box-Cox transformation with a specific lambda for each trait, using maximum likelihood estimation with a grid search implemented in the R package MASS [27], following Box and Cox [28]:

$$f\left( {\mathbf{y}} \right) = \left\{ {\begin{array}{*{20}c} {\frac{{{\mathbf{y}}^{\lambda } - 1}}{\lambda } \left( {\lambda \ne 0} \right)} \\ {\log \left( {\mathbf{y}} \right) (\lambda = 0)} \\ \end{array} } \right.,$$

where $\mathbf{y}$ is the vector of phenotypes for the trait to be transformed and $\lambda$ is the trait specific transformation parameter, which ranged from -3.147 to 5.015.

#### Best linear unbiased prediction models

The models used are summarized in Table 1 and described in the following.

**Table 1:** The BLUP models used in the statistical analyses

Model abbreviation	Model	Random effects	Equation
GBLUP	$\mathbf{y}=\mathbf{X}\mathbf{b}+{\mathbf{Z}}_{g}\mathbf{g}+\mathbf{e}$	SNPs	Equation (1)
TBLUP	$\mathbf{y}=\mathbf{X}\mathbf{b}+{\mathbf{Z}}_{t}\mathbf{t}+\mathbf{e}$	Transcript abundances	Equation (2)
GTBLUP	$\mathbf{y}=\mathbf{X}\mathbf{b}+{\mathbf{Z}}_{g}\mathbf{g}+{\mathbf{Z}}_{t}\mathbf{t}+\mathbf{e}$	SNPs + transcript abundances	Equation (3)
GTCBLUP / GTCBLUPi	$\mathbf{y}=\mathbf{X}\mathbf{b}+{\mathbf{Z}}_{g}\mathbf{g}+{{\mathbf{Z}}_{c}\mathbf{t}}_{c}+\mathbf{e}$	SNPs + conditioned transcript abundances	Equation (4)

$\mathbf{y}$: vector of phenotypes (Box-Cox transformed, scaled, and centered); $\mathbf{X}$: incidence matrix for fixed effects; $\mathbf{b}$: vector of fixed effects (test day); $\mathbf{Z}$: incidence matrix for random effects; $\mathbf{g}$, $\mathbf{t}$: vectors of random effects based on genomic or transcriptomic similarity, respectively; ${\mathbf{t}}_{c}$: vector of random effects based on transcriptomic data conditioned on genetic effects to remove shared variation; $\mathbf{e}$: vector of random residuals

#### GBLUP

The first model used was the following GBLUP model:

1 $${\mathbf{y}} = {\mathbf{Xb}} + {\mathbf{Z}}_{g} {\mathbf{g}} + {\mathbf{e}},$$

where $\mathbf{y}$ is the vector of the Box-Cox transformed, scaled, and centered (mean = 0, SD = 1) phenotypes for a trait, $\mathbf{b}$ is the vector of fixed test day effects, and $\mathbf{X}$ is the associated incidence matrix, ${\mathbf{Z}}_{g}$ is an incidence matrix for $\mathbf{g}$, and $\mathbf{g}$ is an $n$-vector of random additive genetic effects, with $n$ the number of animals, assumed to be distributed as $\mathbf{g} \sim N\left(0,\mathbf{G}{\sigma }_{g}^{2}\right)$, where ${\sigma }_{g}^{2}$ is the additive genetic variance and $\mathbf{G}$ is the additive genomic relationship matrix. Matrix $\mathbf{G}$ was computed following the first method described by VanRaden [29] as $\mathbf{G}=\frac{\mathbf{Z}{\mathbf{Z}}^{\text{T}}}{\sum_{j}2{p}_{j}(1-{p}_{j})}$, where $\mathbf{Z}$ is an $n\times m$ matrix with $m$ the number of centered genotype codes and ${p}_{j}$ is the allele frequency of the reference allele at SNP $j$. Finally, $\mathbf{e}$ is the vector of random residuals, assumed to follow a normal distribution as $\mathbf{e} \sim N\left(0,\mathbf{I}{\sigma }_{e}^{2}\right)$, where ${\sigma }_{e}^{2}$ is the residual variance and $\mathbf{I}$ is an identity matrix.

#### TBLUP

To evaluate the predictive ability of transcriptomic data, we used two transcriptomic BLUP (TBLUP) models. These models are similar to the GBLUP model, but instead of using SNP genotypes to evaluate animal relationships, transcript abundances were used. One TBLUP model was constructed using miRNA data and the other was based on mRNA data. Both TBLUP models had the following form:

2 $${\mathbf{y}} = {\mathbf{Xb}} + {\mathbf{Z}}_{t} {\mathbf{t}} + {\mathbf{e}},$$

where ${\mathbf{Z}}_{t}$ is an incidence matrix and $\mathbf{t}$ is an $n$-vector of random transcript-level effects, assumed to be distributed as $\mathbf{t} \sim N\left(0,\mathbf{T}{\sigma }_{t}^{2}\right)$, where ${\sigma }_{t}^{2}$ is the gene transcript variance, and $\mathbf{T}$ is the transcriptomic relationship matrix. Matrix $\mathbf{T}$ was build as $\mathbf{T}=\frac{\mathbf{W}{\mathbf{W}}^{\text{T}}}{k}$, where $\mathbf{W}$ is the $n\times k$ matrix of pre-corrected transcript abundances, using either the miRNA or the mRNA data, and $k$ is the number of the transcripts. All transcript abundances were Box-Cox transformed and scaled and centered, using the same procedure as described above for the phenotypes. The transcript data were pre-corrected using a mixed linear model accounting for the fixed effects of test day, plate, sex, and the housekeeping genes. The plate represents the technical component for miRNA and mRNA quantification. Housekeeping genes were used as reference genes to account for similar RNA input. The remaining terms of Eq. (2) are as defined in Eq. (1).

#### GTBLUP

To integrate genomic and transcriptomic information into a single model, we constructed a GTBLUP model that treated $\mathbf{g}$ and $\mathbf{t}$ as independent random effects:

3 $${\mathbf{y}} = {\mathbf{Xb}} + {\mathbf{Z}}_{g} {\mathbf{g}} + {\mathbf{Z}}_{t} {\mathbf{t}} + {\mathbf{e}},$$

where all the terms are as defined above in Eq. (1) and (2). These models were applied to either the miRNA or the mRNA transcript abundance data. Joint consideration of miRNA and mRNA data in a BLUP model was not attempted, as strong interactions or covariances would have to be included as additional parameters, which exceeded the scope of this paper.

#### GTCBLUPi

The GTCBLUP model of Perez et al. [11] is similar to the GTBLUP model (Eq. (3)), but avoids the assumption that the random vectors $\mathbf{g}$ and $\mathbf{t}$ are independent, which they are not because transcript levels are partly heritable. Thus, instead of using $\mathbf{t}$ directly in the mixed linear model, Perez et al. [11] replaced $\mathbf{t}$ with ${\mathbf{t}}_{c}=\mathbf{t}-{\mathbf{t}}_{g},$ where ${\mathbf{t}}_{g}={\mathbf{S}}_{\lambda }\mathbf{t}$ is the heritable part of the vector $\mathbf{t}$ with transcript-level effects. Based on this, we provide a more formal derivation of the GTCBLUP model.

The $n\times n$ matrix ${\mathbf{S}}_{\lambda }$ (as defined later) models the heritable part of the gene transcripts. This results in:

$${\mathbf{t}}_{c} = {\mathbf{t}} - {\mathbf{t}}_{g} = {\mathbf{I}}_{n} { }{\mathbf{t}} - {\mathbf{S}}_{\lambda } {\mathbf{t}} = \left( {{\mathbf{I}}_{n} - {\mathbf{S}}_{\lambda } } \right){\mathbf{t}} = \left( {{\mathbf{I}}_{n} - {\mathbf{S}}_{\lambda } } \right){\mathbf{W}} {{\varvec{\uptau}}} = {\mathbf{W}}_{c} {{\varvec{\uptau}}},$$

where ${\varvec{\uptau}}$ is the *k*-vector of transcript-level effects for all transcripts, ${\mathbf{W}}_{c}=\left({\mathbf{I}}_{n}-{\mathbf{S}}_{\lambda }\right)\mathbf{W}$, where $\mathbf{W}$ is the matrix of pre-corrected transcript abundances, and ${\mathbf{I}}_{n}$ is the identity matrix of dimension $n\times n$. We assume that $\text{var}\left({\varvec{\uptau}}\right)=\frac{{\sigma }_{t}^{2}}{k}{\mathbf{I}}_{n}$, where ${\sigma }_{t}^{2}$ is the variance of the gene transcript effects. Therefore, the covariance matrix of the non-heritable transcript-level effects ${\mathbf{t}}_{c}$ is:

$${\text{Var}}\left( {{\mathbf{t}}_{c} } \right) = {\text{Var}}\left( {{\mathbf{W}}_{c} {{\varvec{\uptau}}}} \right) = {\mathbf{W}}_{c} {\text{Var}}\left( {{\varvec{\uptau}}} \right){\mathbf{W}}_{c}^{{\text{T}}} = {\mathbf{W}}_{c} \frac{{\sigma_{t}^{2} }}{k}{\mathbf{I}}_{n} {\mathbf{W}}_{c}^{{\text{T}}} = \frac{{\sigma_{t}^{2} }}{k}{\mathbf{W}}_{c} {\mathbf{W}}_{c}^{{\text{T}}} = \sigma_{t}^{2} { }{\mathbf{T}}_{c} ,$$

where ${\mathbf{T}}_{c}=\frac{{\mathbf{W}}_{c}{\mathbf{W}}_{c}^{\text{T}}}{k}$. Thus, the following mixed linear model can be used to estimate variance components:

4 $${\mathbf{y}} = {\mathbf{Xb}} + {\mathbf{Z}}_{g} {\mathbf{g}} + {\mathbf{Z}}_{c} {\mathbf{t}}_{c} + {\mathbf{e}},$$

where random vector ${\mathbf{t}}_{c} \sim N\left(0,{{\mathbf{T}}_{c}\sigma }_{{t}_{c}}^{2}\right)$ contains the non-heritable part of the transcript-level effects, and ${\mathbf{Z}}_{c}$ is the corresponding incidence matrix. The variance explained by the non-heritable transcript-level effects can then be approximated as: ${\widetilde{\sigma }}_{{t}_{c}}^{2}={\sigma }_{{t}_{c}}^{2}\text{mean}\left(\text{diag}\left({\mathbf{T}}_{c}\right)\right).$

Suppose that the vector $\mathbf{t}$ of centered transcript-level effects on a phenotype of interest is known and used to estimate the $m$-vector $\widetilde{{\varvec{\upgamma}}}$ of SNP effects. Since $\mathbf{t}$ has a mean zero, $\widetilde{{\varvec{\upgamma}}}$ can be estimated in a model without an intercept, as: $\mathbf{t}=\mathbf{Z}\widetilde{{\varvec{\upgamma}}}+\widetilde{\mathbf{e}},$ with $\widetilde{{\varvec{\upgamma}}} \sim N\left(0,\mathbf{I}\frac{{\widetilde{\sigma }}_{g}^{2}}{\widetilde{m}}\right)$, $\widetilde{m}=\sum_{j}2{p}_{j}(1-{p}_{j})$, and $\widetilde{\mathbf{e}} \sim N\left(0,{\mathbf{I}\widetilde{\sigma }}_{e}^{2}\right)$. The BLUP-estimate of $\widetilde{{\varvec{\upgamma}}}$ is: ${\widehat{{\varvec{\upgamma}}}}_{t}={\left({\mathbf{Z}}^{\text{T}}\mathbf{Z}+\lambda {\mathbf{I}}_{m}\right)}^{-1}{\mathbf{Z}}^{\text{T}}\mathbf{t}$, with $\lambda =\frac{{\widetilde{\sigma }}_{e}^{2}}{\frac{{\widetilde{\sigma }}_{g}^{2}}{\widetilde{m}}}=\widetilde{m}\frac{{\widetilde{\sigma }}_{e}^{2}}{{\widetilde{\sigma }}_{g}^{2}}$ and ${\mathbf{I}}_{m}$ is an $m\times m$ identity matrix. Thus, the BLUP estimate of $\mathbf{t}$ obtained from genomic information is: ${\mathbf{t}}_{g}=\mathbf{Z}{\widehat{{\varvec{\upgamma}}}}_{t}=\mathbf{Z}{\left({\mathbf{Z}}^{\text{T}}\mathbf{Z}+\lambda {\mathbf{I}}_{m}\right)}^{-1}{\mathbf{Z}}^{\text{T}}\mathbf{t}={\mathbf{S}}_{\lambda }\mathbf{t}$. Consequently, ${\mathbf{S}}_{\lambda }$ is defined as: ${\mathbf{S}}_{\lambda }=\mathbf{Z}{\left({\mathbf{Z}}^{\text{T}}\mathbf{Z}+\lambda {\mathbf{I}}_{m}\right)}^{-1}{\mathbf{Z}}^{\text{T}}.$ Inserting ${\mathbf{S}}_{\lambda }$ in the formula for ${\mathbf{W}}_{c}$ results in:

5 $${\mathbf{W}}_{c} = \left( {{\mathbf{I}}_{n} - {\mathbf{Z}}\left( {{\mathbf{Z}}^{{\text{T}}} {\mathbf{Z}} + {\mathbf{I}}_{m} \lambda } \right)^{ - 1} {\mathbf{Z}}^{{\text{T}}} } \right){\mathbf{W}}.$$

Because $${\sigma }_{t}^{2}={\widetilde{\sigma }}_{g}^{2}+{\widetilde{\sigma }}_{e}^{2}$$,

$$\lambda = \tilde{m}\frac{{\tilde{\sigma }_{e}^{2} }}{{\tilde{\sigma }_{g}^{2} }} = \tilde{m}\frac{{\sigma_{t}^{2} - \tilde{\sigma }_{g}^{2} }}{{\tilde{\sigma }_{g}^{2} }} = \tilde{m}\left( {\frac{{\sigma_{t}^{2} }}{{\tilde{\sigma }_{g}^{2} }} - 1} \right) = \tilde{m}\left( {\frac{1}{{\tilde{h}_{t}^{2} }} - 1} \right),$$

where ${\widetilde{h}}_{t}^{2}=\frac{{\widetilde{\sigma }}_{g}^{2}}{{\sigma }_{t}^{2}}$ is the heritability of the transcript-level effects. Since this heritability is not known and cannot easily be estimated together with all parameters in the model, parameter $\lambda$ was estimated iteratively by testing different values of ${\widetilde{h}}_{t}^{2}$ (in steps of 0.01) and the value of ${\widetilde{h}}_{t}^{2}$ that maximized the log-likelihood of Eq. (4) was selected. The iteratively estimated values of ${\widetilde{h}}_{t}^{2}$ for a particular trait for the miRNA and mRNA transcript-level effects used in calculating $\lambda$ are shown in Table 2. The log-likelihood of Eq. (4) as a function of ${\widetilde{h}}_{t}^{2}$ can be found in the Additional file 1: Figures S1 and S2.

**Table 2:** Estimates of heritabilities of transcript-level effects (${\widetilde{h}}_{t}^{2}$) on phenotype traits obtained by the iterative maximum log-likelihood procedure

Traits^a^	PU	BWG	FI	FCR	TA	CaU
${\widetilde{h}}_{t}^{2}$(miRNA)	0.25	0.20	0.14	0.64	0.11	0.18
${\widetilde{h}}_{t}^{2}$(mRNA)	0.01	0.09	0.08	0.02	0.01	0.11

^a^*PU*: P utilization, *BWG*: Body weight gain, *FI*: Feed intake, *FCR*: Feed conversion ratio, *TA*: Tibia ash, *CaU*: Ca utilization

Note that Perez et al. [11] sed a different form for $\lambda =m\frac{{\sigma }_{e}^{2}}{{\sigma }_{g}^{2}}=m\left(\frac{1}{{h}^{2}}-1\right)$, which was not entirely justified. Thus, to avoid confusion between the models, we refer to our adapted version as GTCBLUPi.

In GTCBLUPi, we use Eq. (5) but when many SNPs are included, the matrix ${\mathbf{Z}}^{\text{T}}\mathbf{Z}$ becomes very large, and thus the computational capacity to form matrix ${\mathbf{W}}_{c}$ (Eq. (5)) can be a challenge. Using the Woodbury matrix identity [30] and realizing that ${\mathbf{Z}}^{\text{T}}\mathbf{Z}={\mathbf{Z}}^{\text{T}}{\mathbf{I}}_{n}\mathbf{Z}$, we can, however, re-write ${\left({\mathbf{Z}}^{\text{T}}\mathbf{Z}+{\mathbf{I}}_{m}\lambda \right)}^{-1}$ as:

$$\begin{gathered} \left( {{\mathbf{Z}}^{{\text{T}}} {\mathbf{Z}} + {\mathbf{I}}_{m} \lambda } \right)^{ - 1} \, = \,{\mathbf{I}}_{m} \lambda^{ - 1} - {\mathbf{I}}_{m} \lambda^{ - 1} {\mathbf{Z}}^{{\text{T}}} \left( {{\mathbf{I}}_{n} + {\mathbf{ZI}}_{m} \lambda^{ - 1} {\mathbf{Z}}^{{\text{T}}} } \right)^{ - 1} {\mathbf{ZI}}_{m} \lambda^{ - 1} \hfill \\ \quad \quad \quad \quad \quad \quad = {\mathbf{I}}_{m} \lambda^{ - 1} - \lambda^{ - 1} {\mathbf{Z}}^{{\text{T}}} \left( {{\mathbf{I}}_{n} + {\mathbf{ZZ}}^{{\text{T}}} \lambda^{ - 1} } \right)^{ - 1} {\mathbf{Z}}\lambda^{ - 1} \hfill \\ \end{gathered}$$

Note that $\mathbf{G}$ ($n\times n$), following VanRaden method 1, is defined as: $\mathbf{G}=\frac{{\mathbf{Z}\mathbf{Z}}^{\text{T}}}{\sum_{j}2{p}_{j}(1-{p}_{j})}$. Inserting ${\mathbf{Z}\mathbf{Z}}^{\text{T}}=\mathbf{G}\sum_{j}2{p}_{j}(1-{p}_{j})$ into the above formula gives:

$$\left( {{\mathbf{Z}}^{{\text{T}}} {\mathbf{Z}} + {\mathbf{I}}_{m} \lambda } \right)^{ - 1} = {\mathbf{I}}_{m} \lambda^{ - 1} - \lambda^{ - 1} {\mathbf{Z}}^{{\text{T}}} \left( {{\mathbf{I}}_{n} + {\mathbf{G}}\sum\nolimits_{j} {2p_{j} \left( {1 - p_{j} } \right)\lambda^{ - 1} } } \right)^{ - 1} {\mathbf{Z}}\lambda^{ - 1}$$

Putting this into Eq. (5), we get:

$$\begin{gathered} {\mathbf{W}}_{c} \, = \,\left( {{\mathbf{I}}_{n} - {\mathbf{Z}}\left( {{\mathbf{I}}_{m} \lambda^{ - 1} - \lambda^{ - 1} {\mathbf{Z}}^{{\text{T}}} \left( {{\mathbf{I}}_{n} + {\mathbf{G}}\sum\nolimits_{j} {2p_{j} \left( {1 - p_{j} } \right)\lambda^{ - 1} } } \right)^{ - 1} {\mathbf{Z}}\lambda^{ - 1} } \right){\mathbf{Z}}^{{\text{T}}} } \right){\mathbf{W}} \hfill \\ \quad \,\, = \,\left( {{\mathbf{I}}_{n} - {\mathbf{ZI}}_{m} {\mathbf{Z}}^{{\text{T}}} \lambda^{ - 1} + \left( {{\mathbf{ZZ}}^{{\text{T}}} \lambda^{ - 1} \left( {{\mathbf{I}}_{n} + {\mathbf{G}}\sum\nolimits_{j} {2p_{j} \left( {1 - p_{j} } \right)\lambda^{ - 1} } } \right)^{ - 1} {\mathbf{ZZ}}^{{\text{T}}} \lambda^{ - 1} } \right)} \right){\mathbf{W}} \hfill \\ \quad \,\, = \,\left( {{\mathbf{I}}_{n} - {\mathbf{G}}\sum\nolimits_{j} {2p_{j} \left( {1 - p_{j} } \right)\lambda^{ - 1} } \, + \,\left( {{\mathbf{G}}\sum\nolimits_{j} {2p_{j} \left( {1 - p_{j} } \right)\lambda^{ - 1} } \,\left( {{\mathbf{I}}_{n} + {\mathbf{G}}\sum\nolimits_{j} {2p_{j} \left( {1 - p_{j} } \right)\lambda^{ - 1} } } \right)^{ - 1} {\mathbf{G}}\sum\nolimits_{j} {2p_{j} \left( {1 - p_{j} } \right)\lambda^{ - 1} } } \right)} \right){\mathbf{W}} \hfill \\ \end{gathered}$$

Defining ${\mathbf{G}}^{\boldsymbol{*}}=\mathbf{G}\sum_{j}2{p}_{j}(1-{p}_{j}){\lambda }^{-1}$, this simplifies to:

6 $${\mathbf{W}}_{c} = \left( {{\mathbf{I}}_{n} - {\mathbf{G}}^{\user2{*}} + \left( {{\mathbf{G}}^{\user2{*}} \left( {{\mathbf{I}}_{n} + {\mathbf{G}}^{\user2{*}} } \right)^{ - 1} {\mathbf{G}}^{\user2{*}} } \right)} \right){\mathbf{W}}.$$

Given that the matrix inversion in Eq. (5) and (6) are likely the time-limiting steps to compute, we advise to use Eq. (5) if the number of animals is larger than the number of SNPs, and Eq. (6) if the number of SNPs is larger than the number of animals.

#### Variance components

To quantify how much of the phenotypic variance of the phenotypic traits can be explained by SNP genotypes and transcript abundances, we estimated variance components using the BLUP models in Table 1. To explain the proportion of individual variance components to phenotypic variance, the respective variance components were divided by the phenotypic variance. For the GTCBLUPi model, this means that the proportion of variance explained by the SNP genotypes (i.e. heritability) was calculated as ${h}^{2}=\frac{{\sigma }_{g}^{2}}{{\sigma }_{g}^{2}+{\widetilde{\sigma }}_{{t}_{c}}^{2}+{\sigma }_{e}^{2}}$, and the proportion of variance explained by the conditioned gene transcripts as ${{t}_{c}}^{2}=\frac{{\widetilde{\sigma }}_{{t}_{c}}^{2}}{{\sigma }_{g}^{2}+{\widetilde{\sigma }}_{{t}_{c}}^{2}+{\sigma }_{e}^{2}}$. The same was applied to the variance components of the other BLUP models.

For each pair of nested models, we evaluated whether the model with two explanatory variables (e.g. GTBLUP) resulted in a significantly better fit to the data than the model with one explanatory variable (GBLUP or TBLUP). The fits of the nested models to the data were compared using likelihood ratio tests as $D=2\left[\text{log}\left({L}_{2}\right)-\text{log}\left({L}_{1}\right)\right]$, where ${L}_{2}$ is the likelihood of the full model and ${L}_{1}$ the likelihood of the model without the corresponding random effect to be tested [31]. To compare the fit of non-nested models, we calculated the Akaike information criterion (AIC) for each model [31].

#### Trait phenotype prediction

To evaluate the suitability of the BLUP models for predicting the phenotype of traits, we used random cross-validation with a total of 500 repetitions. In each run, 80% of the animals were randomly selected as the reference population and the genomic and/or transcriptomic effects were estimated. The remaining 20% of the animals in each replicate formed the validation population, for which the animal effects were predicted, based on the estimates from the reference population. Prediction accuracy was defined as the average Pearson correlation between the predicted animal effects and observed phenotypes in the validation population, pre-corrected for fixed test day effects, across 500 repetitions of a random 80/20 split. Although each individual will appear multiple times in the training and test sets across replicates, the replicates are not completely independent, the average accuracy is considered a robust estimate of the model performance.

To calculate the approximate 95% confidence intervals of the prediction accuracy, we applied Fisher’s z-transformation [32] to the correlation estimates: $z=\frac{1}{2}ln(\frac{1+\mathbf{r}}{1-\mathbf{r}})$. After calculating the confidence intervals for the Fisher’s z-values, they were transformed back to restore the original correlation range by applying the inverse Fisher’s z-transformation: $r=\frac{{e}^{2z}-1}{{e}^{2z}+1\boldsymbol{}}$.

To generate the 480 F2 birds of this study, a total of 10 F1 males were mated with 20 F1 females, with each male paired with two females, without rotation. To assess the stability of predictive accuracy of the phenotypes in populations with lower relatedness between reference and validation animals, we used the F2 family design to divide the animals into reference and validation population. Accordingly, all F2 animals were grouped based on their paternal half-sib structure ($n=10$), meaning that all F2 half- and full-sib families were clustered together. In each run, 80% of the family groups ($n=8$) were used as the reference population and the remaining 20% ($n=2$) as the validation population. This grouping ensured that the number of animals in the validation and reference population was comparable to the random cross-validation. To ensure robustness, repetitions were employed such that each of the 45 $$(\left(\begin{array}{c}10\\ 2\end{array}\right)=45)$$ possible distribution of the groups occurred once. The mean prediction accuracies and their 95% confidence intervals were calculated as described above.

To test for significant differences between the prediction accuracies of the BLUP models, we conducted pairwise correlation comparisons across the same repetitions, following Schrauf et al. [33]. In each repetition, the same animals were used for the validation and reference population for each BLUP model applied. For each repetition, we calculated the difference in accuracy between the models (i.e. the difference of the Pearson correlations between the predicted animal effects and observed phenotypes, pre-corrected for fixed test day effects) and computed the mean and 95% confidence intervals for the 500 or 45 differences in accuracies using the previously described method. A significant difference was declared when the confidence intervals did not include zero.

#### Transcriptomic trait correlations

The genetic and phenotypic correlations of the phenotyped traits were published previously [20, 24], as mentioned above. To estimate the correlations of transcriptomic animal effects between the traits, the TBLUP models were extended to bivariate models. To determine whether the correlations differ significantly from zero, we tested their significance using likelihood ratio tests, as explained above, with the hypothesis ${{H}_{0}:r}_{t1,t2}=0$ against ${{H}_{1}: r}_{t1,t2}\ne 0$. In these tests, ${L}_{2}$ represents the likelihood of the full bivariate model and ${L}_{1}$ the likelihood of the bivariate model with the transcriptomic correlation fixed at zero.

#### Estimating the effect of individual transcript abundances on phenotypes

To infer the transcriptomic architecture of the investigated traits by estimating the effect of individual transcript abundances on phenotypes, the transcriptomic animal effects from Eq. (2) of the TBLUP models, were back-solved following Vollmar et al. [34] to obtain the BLUP of the $k$-dimensional vector of estimated transcript-level effects as:

7 $${\hat{\mathbf{u}}} = \frac{1}{k}{\mathbf{W}}^{{\text{T}}} {\mathbf{T}}^{ - 1} {\hat{\mathbf{t}}},$$

with the corresponding variance:

$${\text{Var}}\left( {{\hat{\mathbf{u}}}} \right) = {\text{Var}}\left( {\frac{1}{k}{\mathbf{W}}^{{\text{T}}} {\mathbf{T}}^{ - 1} {\hat{\mathbf{t}}}} \right) = \frac{1}{{k^{2} }}{\mathbf{W}}^{{\text{T}}} {\mathbf{T}}^{ - 1} {\text{Var}}\left( {{\hat{\mathbf{t}}}} \right){\mathbf{T}}^{ - 1} {\mathbf{W}},$$

where $\widehat{\mathbf{t}}$ is the $n$-dimensional vector of estimated animal transcriptomic effects obtained from Eq. (2).

Since $\text{Cov}\left(\mathbf{t},\widehat{\mathbf{t}}\right)=\text{Var}\left(\widehat{\mathbf{t}}\right)$, the predictor error variance (PEV) of $\widehat{\mathbf{t}}$ from Eq. (2) is equal to:

$${\text{PEV}}\left( {{\hat{\mathbf{t}}}} \right) = {\text{Var}}\left( {{\mathbf{t}} - {\hat{\mathbf{t}}}} \right) = {\text{Var}}\left( {\mathbf{t}} \right) - {\text{Var}}\left( {{\hat{\mathbf{t}}}} \right) = {\mathbf{C}}^{tt} ,$$

where ${\mathbf{C}}^{tt}$ is the inverse of the coefficient matrix of the mixed model equations, following Gualdrón Duarte et al. [35], based on Henderson [36]. The incidence matrix ${\mathbf{Z}}_{t}$ of $\mathbf{t}$ is an identity matrix in our case, so was omitted from the following derivation:

$${\mathbf{C}}^{tt} = \sigma_{e}^{2} \left( {{\mathbf{I}} - {\mathbf{X}}\left( {{\mathbf{X}}^{{\text{T}}} {\mathbf{X}}} \right)^{ - 1} {\mathbf{X}}^{{\text{T}}} + {\mathbf{T}}^{ - 1} \lambda } \right)^{ - 1} ,\,{\text{with}}\,\lambda = \frac{{\sigma_{e}^{2} }}{{\sigma_{t}^{2} }}.$$

Thus, we have, $\text{Var}\left(\widehat{\mathbf{t}}\right)=\text{Var}\left(\mathbf{t}\right)-{\mathbf{C}}^{uu}=\mathbf{T}{\sigma }_{t}^{2}-{\mathbf{C}}^{tt}$. Then, all elements were combined and the unknown parameters were replaced with their derived expressions, giving:

$$\begin{gathered} {\text{Var}}\left( {{\hat{\mathbf{u}}}} \right)\, = \,\frac{1}{{k^{2} }}{\mathbf{W}}^{{\text{T}}} {\mathbf{T}}^{ - 1} {\text{Var}}\left( {{\hat{\mathbf{t}}}} \right){\mathbf{T}}^{ - 1} {\mathbf{W}} \hfill \\ \quad \quad \quad = \,\frac{1}{{k^{2} }}{\mathbf{W}}^{{\text{T}}} {\mathbf{T}}^{ - 1} \left( {{\mathbf{T}}\sigma_{t}^{2} - {\mathbf{C}}^{tt} } \right) {\mathbf{T}}^{ - 1} {\mathbf{W}} \hfill \\ \end{gathered}$$

This test statistic for the transcript-level effects $\widehat{\mathbf{u}}$ was derived following Gualdrón Duarte et al. [35] and Aguilar et al. [37] but using transcript rather than SNP effects. First, the estimated effect ${\widehat{\text{u}}}_{j}$ of the $j$-th transcript was standardized as:

$$RNA_{j} = \frac{{{\hat{\text{u}}}_{j} }}{{\sqrt {{\text{Var}}\left( {{\hat{\text{u}}}_{j} } \right)} }}.$$

The absolute value $\left|{RNA}_{j}\right|$ was used as the test statistic for the hypothesis ${H}_{0}: |{\widehat{\text{u}}}_{j}|=0$ against ${H}_{1}: |{\widehat{\text{u}}}_{j}|>0$. Since ${RNA}_{j}$ has approximately a normal distribution with mean zero and standard deviation 1, p-values for this test can be computed as:

$$p - value_{j} = 2\left( {1 - {\Phi }\left( {\left| {RNA_{j} } \right|} \right)} \right),$$

where $\Phi \left(\text{x}\right)$ is the cumulative density function of the normal distribution for the random variable $\text{x}$.

## Results

### Variance components and model fit

**Fig. 1:**
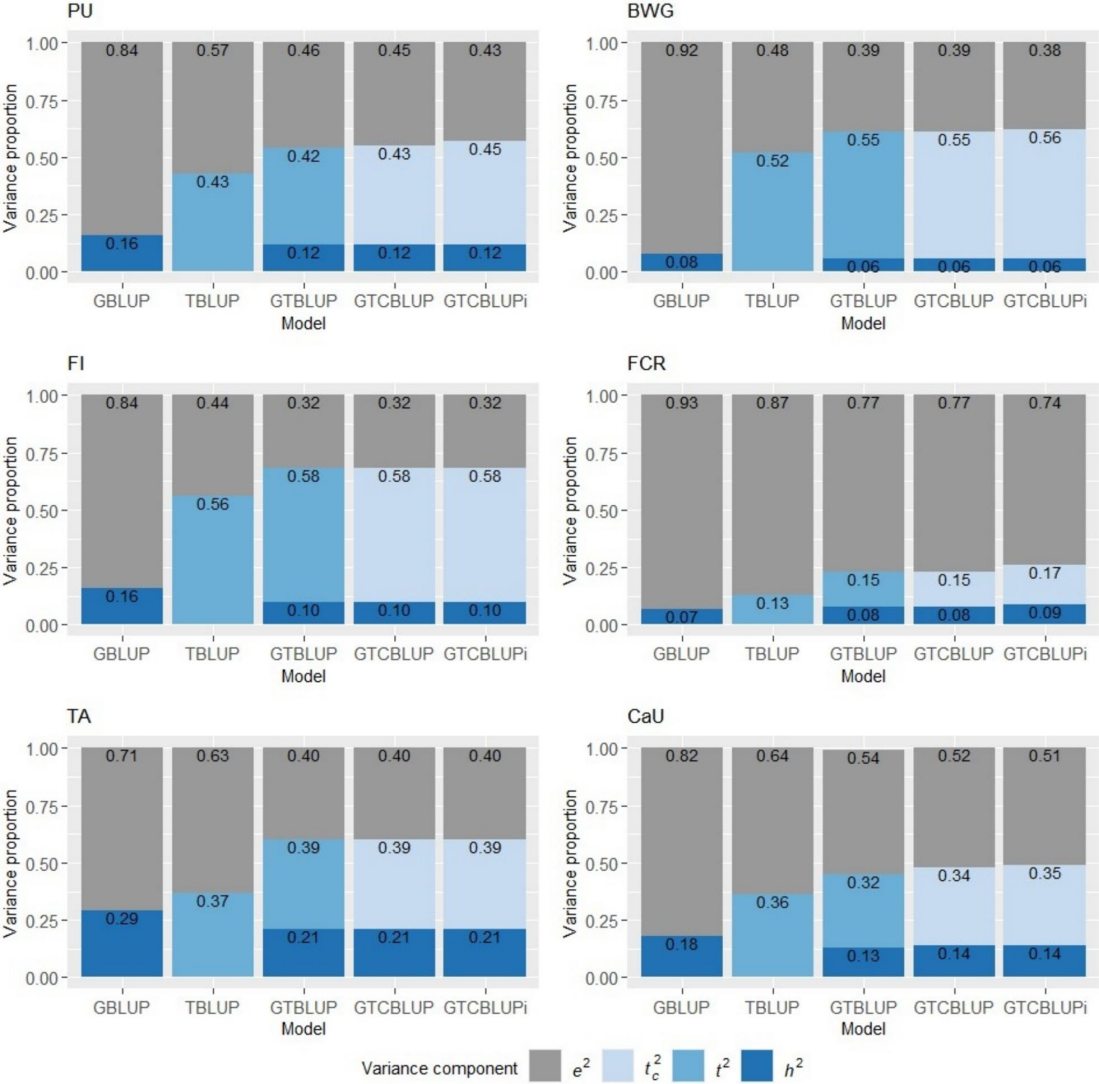
Estimates of the proportions of variance explained by SNP genotypes (${h}^{2}$), miRNA transcript abundances (${{t}_{c}}^{2}$ / ${t}^{2}$) and the residual variance (${e}^{2}$) for the traits P utilization (PU), body weight gain (BWG), feed intake (FI), feed conversion ratio (FCR), tibia ash (TA), and Ca utilization (CaU), estimated with GBLUP, TBLUP, GTBLUP, GTCBLUP, and GTCBLUPi. For a description of the models, see Table 1

The variance components estimated using the different BLUP models are presented in bar plots for each trait, arranged in ascending order of model complexity, from GBLUP to GTCBLUPi. Estimates of the variance components are shown as proportions of the phenotypic variance, for miRNA data in Fig. 1, and for mRNA data in Additional file 1: Figure S3. Additional file 2: Table S1 lists all estimates or variance components and their corresponding standard errors for all models and traits.

Estimates of genomic heritabilities (*h*^2^) of the six traits obtained with GBLUP ranged from 0.07 (FCR) to 0.29 (TA). The estimate of the proportion of transcriptomic variance (*t*^2^) based on TBLUP varied from 0.13 (FCR) to 0.56 (FI) using miRNA data, and from 0.21 (CaU) to 0.33 (FCR) using mRNA data. Note that the estimate of *t*^2^ was higher than the estimate of *h*^2^ for all traits, except for TA using mRNA data. For the traits PU, BWG, FCR, and CaU (Fig. 1), the estimate of the proportion of residual variance (*e*^2^) using miRNA was lowest for the GTCBLUPi model compared to the others. The pattern of decreasing estimates of *e*^2^ followed the complexity of the models (from GBLUP to GTCBLUPi). This was associated with higher estimates of ${t}^{2}$ for the traits PU, BWG, and FCR and higher estimates of ${h}^{2}$ for trait FCR. For FI and TA, the variance proportion estimates were consistent across models that incorporated both SNPs and transcripts (GTBLUP, GTCBLUP, and GTCBLUPi), while for CaU estimates of ${h}^{2}$ and ${t}^{2}$ were smaller compared to those obtained with GBLUP and TBLUP.

Models with transcriptomic miRNA information exhibited higher estimates of phenotypic variance (sum of the model-specific estimated variance components) compared to the GBLUP models (see Additional file 2: Table S1). The variance estimates differed only marginally between the GTBLUP, GTCBLUP, and GTCBLUPi models. The estimates for mRNA data differed slightly from those for miRNA data. While the combined modeling of SNPs and transcripts explained a greater proportion of the phenotypic variance, it led to slightly lower ${h}^{2}$ estimates for the traits PU, FCR, TA, and CaU, and slightly lower ${t}^{2}$ estimates for PU and CaU compared to GLBUP and TBLUP. For BWG and FI, estimates for both ${h}^{2}$ and ${t}^{2}$ remained unchanged, while for FCR, the estimate of ${t}^{2}$ stayed the same. A strong reduction in the estimate of ${h}^{2}$ was observed for FCR when SNPs and transcripts were modeled together, with estimates of ${h}^{2}$ decreasing from 0.07 towards zero.

To assess the model fit of the BLUP models, we calculated the AIC for each model and trait. Using miRNA data, the model fit improved with increasing model complexity, as evident from a decrease in AIC from GBLUP to GTCBLUPi (Table 3). The same pattern was observed using the mRNA data (see Additional file 2: Table S2), except for FCR, for which the TBLUP model had the lowest AIC, and for TA, for which GTBLUP had the lowest AIC. The AIC differences between models were largest between GBLUP and TBLUP for the traits BWG and FI when using miRNA data and for the traits PU, BWG, FI, FCR when using mRNA data. Additionally, the largest differences in AIC were found between the models with one explanatory variable (GBLUP and TBLUP) and those with two explanatory variables (GTBLUP, GTCBLUP, and GTCBLUPi) for the traits PU, TA, and CaU when using miRNA data and for the traits TA and CaU when using mRNA data. Among the models with two explanatory variables, AICs decreased from GTBLUP to GTCBLUPi. The likelihood ratio test (see Additional file 2: Table S3) showed that adding the second explanatory variable significantly improved model fit (p < 0.05) in almost all cases.

**Table 3:** Akaike information criterion (AIC) results for each trait for different BLUP models using miRNA transcripts

Trait^a^	GBLUP	TBLUP	GTBLUP	GTCBLUP	GTCBLUPi
PU	482.61^†^	471.50	451.51	449.42	448.29^‡^
BWG	466.40^†^	422.68	414.99	413.11	410.46^‡^
FI	436.49^†^	406.07	383.55	380.79	380.37^‡^
FCR	503.10^†^	496.75	495.73	495.23	491.84^‡^
TA	445.38	456.40^†^	419.39	417.57	417.54^‡^
CaU	478.57	484.55^†^	463.03	461.88	461.64^‡^

^a^*PU* P utilization, *BWG* Body weight gain, *FI* Feed intake, *FCR* Feed conversion ratio, *TA* Tibia ash, *CaU* Ca utilization

^†^indicates the highest AIC value

^‡^indicates the lowest AIC value. For a description of the models, see Table 1

### Prediction accuracy of trait phenotypes

Table 4 presents the results for phenotype prediction using random cross-validation with the miRNA data models. Compared to the GBLUP model, the TBLUP model generally yielded in higher trait prediction accuracies—with the accuracy for BWG even more than doubling (GBLUP: 0.15, TBLUP: 0.38), while a slight reduction was observed for TA (GBLUP: 0.30, TBLUP: 0.26). The accuracy for GBLUP ranged from 0.11 to 0.30, while for TBLUP it ranged from 0.16 to 0.39. When comparing models with both genomic and transcriptomic data as explanatory variables (GTBLUP, GTCBLUP, GTCBLUPi) to those using a single explanatory variable (GBLUP, TBLUP), prediction accuracies were higher for all traits. The accuracies of trait predictions of the models with two explanatory variables were all in a similar range, with slightly higher accuracies for FCR using the GTCBLUPi model (GTBLUP: 0.17, GTCBLUPi: 0.21). For most traits, the confidence intervals of the prediction accuracies did not include zero, except for BWG with the GBLUP model and for FCR with the GBLUP and TBLUP models.

**Table 4:** Phenotype prediction accuracies of the BLUP models with miRNA transcripts for different traits using random cross-validation

Trait^a^	GBLUP		TBLUP		GTBLUP		GTCBLUP		GTCBLUPi	
	Accuracy^b^	95% CI^c^	Accuracy	95% CI	Accuracy	95% CI	Accuracy	95% CI	Accuracy	95% CI
PU	0.24	0.09:0.39	0.32	0.15:0.47	0.39	0.24:0.52	0.39	0.24:0.53	0.40	0.25:0.53
BWG	0.15	−0.04:0.32	0.38	0.21:0.53	0.41	0.24:0.56	0.41	0.25:0.56	0.42	0.25:0.57
FI	0.23	0.06:0.40	0.39	0.22:0.54	0.46	0.31:0.59	0.47	0.31:0.60	0.46	0.31:0.60
FCR	0.11	−0.05:0.26	0.16	−0.01:0.33	0.17	0.01:0.33	0.18	0.01:0.34	0.21	0.03:0.37
TA	0.30	0.14:0.46	0.26	0.08:0.44	0.42	0.26:0.56	0.42	0.26:0.57	0.42	0.26:0.57
CaU	0.27	0.13:0.41	0.28	0.12:0.43	0.36	0.21:0.49	0.36	0.22:0.50	0.36	0.22:0.50

^a^*PU* P utilization, *BWG* Body weight gain, *FI* Feed intake, *FCR* Feed conversion ratio, *TA* Tibia ash, *CaU* Ca utilization

^b^Mean accuracies

^c^95% confidence intervals. For a description of the models, see Table 1

Table 5 shows the prediction accuracies using family-based cross-validation for the models with miRNA data. It shows that the prediction accuracy of all models and traits decreased compared to the prediction accuracies using random cross-validation (Table 4). GBLUP predictions were highly uncertain, with accuracies ranging from 0 to 0.17, and confidence intervals consistently including zero. However, in contrast to GBLUP, models incorporating transcriptomic data, prediction accuracy remained relatively stable, with only slight decreases relative to the random cross-validation results. Moreover, the confidence intervals for PU, BWG, FI, and CaU did not include zero. As with the random cross-validation results, only minor differences in prediction accuracy were observed in the family-based cross-validation between the different models that all incorporated both genomic and transcriptomic information.

**Table 5:** Phenotype prediction accuracies of the BLUP models with miRNA transcripts for different traits using family-based cross-validation

Trait^a^	GBLUP		TBLUP		GTBLUP		GTCBLUP		GTCBLUPi	
	Accuracy^b^	95% CI^c^	Accuracy	95% CI	Accuracy	95% CI	Accuracy	95% CI	Accuracy	95% CI
PU	0.00	−0.18:0.18	0.26	0.05:0.45	0.26	0.08:0.43	0.27	0.10:0.43	0.28	0.11:0.44
BWG	0.08	−0.10:0.26	0.36	0.06:0.60	0.39	0.10:0.62	0.39	0.11:0.63	0.40	0.12:0.63
FI	0.13	−0.05:0.31	0.34	0.04:0.59	0.39	0.11:0.62	0.40	0.13:0.63	0.40	0.13:0.63
FCR	0.01	−0.15:0.16	0.14	−0.06:0.34	0.14	−0.06:0.33	0.15	−0.05:0.33	0.15	−0.08:0.37
TA	0.17	−0.07:0.40	0.18	−0.09:0.42	0.26	−0.02:0.51	0.28	0.00:0.53	0.28	0.00:0.53
CaU	0.02	−0.21:0.24	0.22	0.04:0.40	0.20	0.03:0.36	0.21	0.04:0.37	0.21	0.04:0.37

^a^*PU* P utilization, *BWG* Body weight gain, *FI* Feed intake, *FCR* Feed conversion ratio, *TA* Tibia ash, *CaU* Ca utilization

^b^Mean accuracies

^c^95% confidence intervals. For a description of the models, see Table 1

The prediction accuracies of models using mRNA data were comparable to those using miRNA data, with higher accuracies observed for FCR and TA by using mRNA data. In both cases (miRNA and mRNA), models with two explanatory variables achieved the highest prediction accuracies (Table 4; Additional file 2: Table S4). For the family-based cross-validation (see Additional file 2: Table S5), models incorporating transcriptomic information yielded very similar accuracies as using miRNA (Table 5), with zero not being included in the confidence intervals. Overall, predictive accuracy was lower for the family-based cross-validation compared to random cross-validation, mirroring the pattern with the miRNA data.

**Fig. 2:**
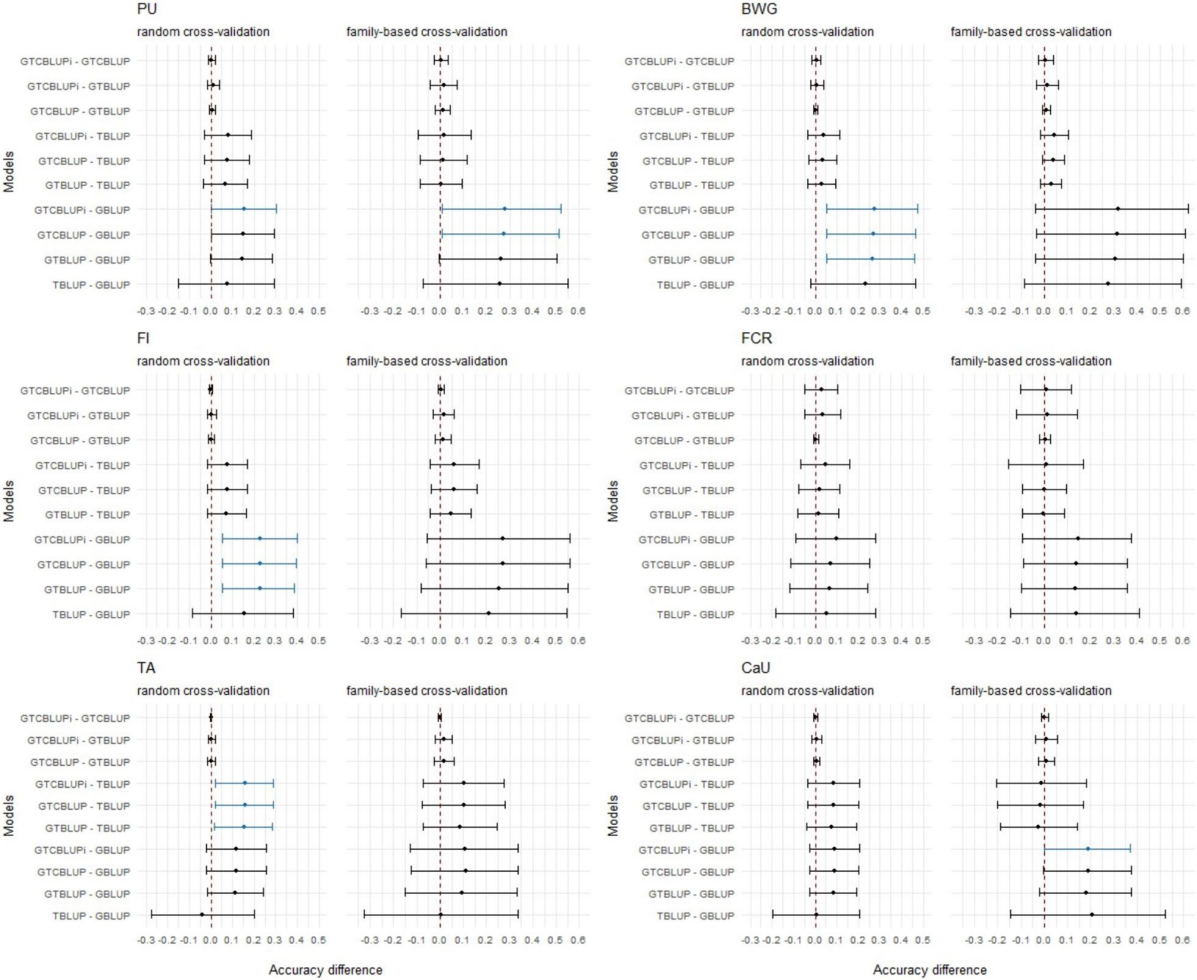
Estimates of differences in accuracy between the different BLUP models using miRNA transcript abundances. The average accuracies of the 500 and 45 differences are displayed as dots, the corresponding 95% confidence intervals as horizontal lines. Differences whose confidence intervals do not include zero are shown in blue. For a description of the models, see Table 1

Figure 2 shows the average differences in accuracy between BLUP models (using miRNA data) across all repetitions, along with the corresponding 95% confidence intervals. If the average difference and its confidence interval do not include zero, a significant difference in the prediction accuracy between the models was indicated. For PU, GTCBLUPi significantly outperformed GBLUP when using random cross-validation, and GTCBLUP and GTCBLUPi had significantly higher prediction accuracies than the GBLUP model when using family-based cross-validation. For the traits BWG and FI, the GTBLUP, GTCBLUP, and GTCBLUPi models significantly outperformed the GBLUP model in trait prediction when using random cross-validation. For TA, the GTBLUP, GTCBLUP, and GTCBLUPi models had significantly higher prediction accuracies than the TBLUP model when using random cross-validation. CaU showed significantly higher prediction accuracy for GTCBLUPi than for GBLUP when using family-based cross-validation. No other significant differences were observed for the miRNA data models.

The average differences in prediction accuracy and the corresponding confidence intervals obtained from the models using mRNA data are shown in Additional file 3: Figure S4. For PU, all models outperformed the GBLUP model when using family-based cross-validation. For BWG and FCR, the GBLUP model exhibited significantly lower accuracies than all other models for both random and family-based cross-validation. The GTBLUP, GTCBLUP, and GTCBLUPi models achieved significantly higher prediction accuracies for FI compared to the GBLUP model for both types of cross-validations. For CaU the GTBLUP, GTCBLUP, and GTCBLUPi models were significantly superior to GBLUP model when using family-based cross-validation. No other significant differences were observed.

### Transcriptomic trait correlations

The results of the bivariate TBLUP models are displayed in Table 6. Estimates of animal transcriptomic trait correlations based on miRNA transcript abundances are shown below the diagonal and those based on mRNA transcript abundances above the diagonal. Most correlation estimates were relatively close to 1 or −1 and differed only slightly between the trait combinations. Almost all correlation estimates were significantly different from zero ($${{H}_{0}:r}_{t1,t2}=0)$$, as indicated by the p-values. For FCR, only negative correlations with the other traits were estimated.

**Table 6:** Estimates of transcriptomic trait correlations based on miRNA (below the diagonal) and mRNA (above the diagonal)

Traits^a^	PU	BWG	FI	FCR	TA	CaU
PU		0.99 (0.02)**	0.99 (0.04)**	−0.61 (0.16)**	1.00 (0.02)**	0.92 (0.05)**
BWG	0.90 (0.06)**		0.93 (0.04)**	−0.78 (0.09)**	0.87 (0.07)**	0.93 (0.07)**
FI	0.95 (0.04)**	1.00 (0.01)**		−0.46 (0.18)*	0.92 (0.05)**	0.97 (0.05)**
FCR	−0.68 (0.22)*	−0.98 (0.05)**	−0.97 (0.09)**		−0.46 (0.20)*	−0.48 (0.21)
TA	1.00 (0.01)**	0.90 (0.06)**	0.95 (0.03)**	−1.00 (0.03)*		0.99 (0.04)**
CaU	0.91 (0.05)**	0.74 (0.12)**	0.84 (0.09)**	−0.19 (0.34)	0.98 (0.03)**	

Standard errors (SE) are shown in parentheses, and asterisks indicate the results of the significance test, showing significant differences from zero (*p < 0.05; **p < 0.01)

^a^*PU* P utilization, *BWG* Body weight gain, *FI* Feed intake, *FCR* Feed conversion ratio, *TA* Tibia ash, *CaU* Ca utilization

### Estimates of the effect of individual transcript abundances on phenotypes

**Fig. 3:**
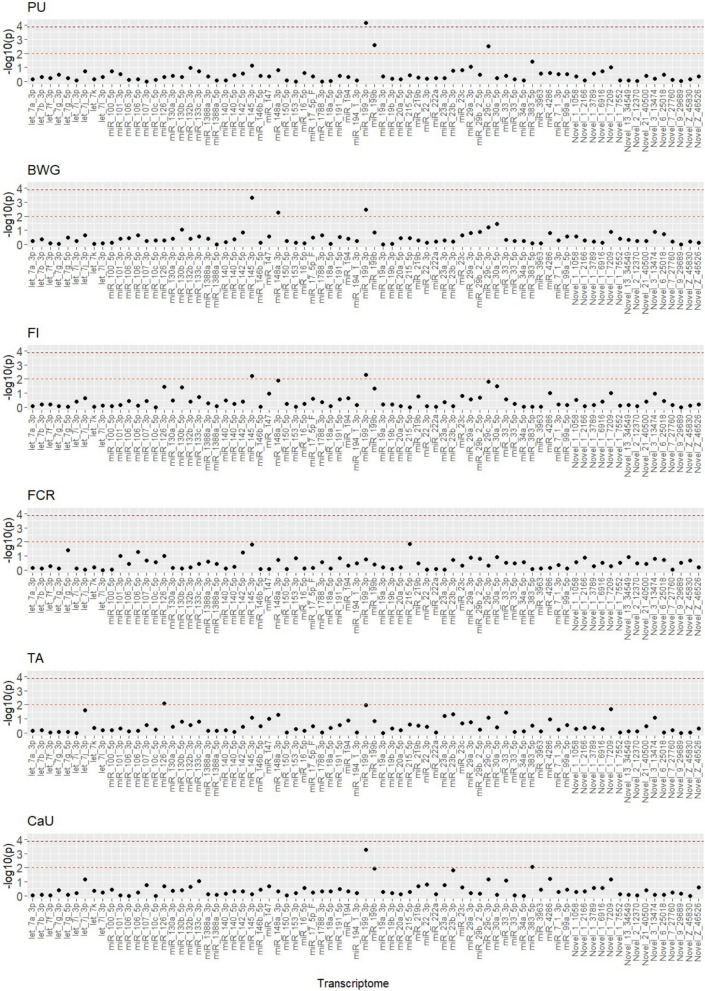
Results of the effect estimation of individual transcript abundances on the phenotypes P utilization (PU), body weight gain (BWG), feed intake (FI), feed conversion ratio (FCR), tibia ash (TA), and Ca utilization (CaU). The −log10(p-values) of the miRNAs are shown. The slight red line (lower) corresponds to the significance level of p = 0.05 and the dark red line (upper) to the Bonferroni-corrected p-value of 0.05

The results of the effect estimation of individual transcript abundances on phenotypes are shown in Fig. 3 for the miRNA data and in Additional file 3: Figure S5 for the mRNA data. We applied two significance thresholds: a nominal significance level of 0.05 and a Bonferroni-corrected threshold of 0.05 to account for multiple testing. Results showed many transcripts with small effects and only a few with large effects. Notably, the miRNA miR_199_3p displayed a visible peak in the Manhatten plot for PU, BWG, FI, TA, and CaU, while miRNA miR_145_3p showed a slight peak for BWG and FI. The mRNA results mirrored these patterns, with several transcripts showing small effects and only a few displaying larger effects. CAV1, in particular, showed a slight peak for PU, FI, TA, and CaU. Note that most of the detected peaks were only nominally significant and should be considered as suggestive.

## Discussion

This study investigated the effects of adding transcript abundances of miRNAs or mRNAs as explanatory variables in genomic prediction models, applied to phenotypic traits of a Japanese quail F2 cross dataset. Several BLUP models were used to compare estimated variances and the predictive ability of genomic and transcriptomic data, with a focus on the GTCBLUPi model, which was developed by Perez et al. [11] and more formally derived and modified here. Additionally, we presented two equivalent approaches to condition the gene transcripts on the genotypes, of which the most efficient one can be determined based on the number of SNPs versus the number of animals. We also estimated transcriptomic trait correlations and effects of individual transcript abundances on the phenotypes to explore the transcriptomic background of the phenotypic traits.

### Variances

The genomic variance, estimated by GBLUP, explained a markedly smaller proportion of the phenotypic variance compared to the transcriptomic variance estimated with TBLUP. Perez et al. [11] and Ehsani et al. [38] also found that transcripts explain more variance than SNPs for performance traits in mice, a finding also observed by Morgante et al. [39] for fitness and behaviour traits in fruit flies, and by Jia et al. [40] for growth traits in meat rabbits.

The models that incorporated both genomic and transcriptomic data, especially the GTCBLUPi model, explained the highest phenotypic variance and had the smallest AICs for most traits. Transcriptomic data provide an additional contribution to the explanation of phenotypic variance that goes beyond genomics. The integration of genomic and transcriptomic data therefore enables a more comprehensive view and improves the explanatory power of complex traits.

Other studies have confirmed that combining genetic and transcriptomic data as independent variables in one model explains more phenotypic variance (e.g. [11, 38]). In contrast, Takagi et al. [41] observed a reduction of genetic variance of more than 55% by modeling the genetic and transcriptomic information simultaneously in one model (modeled as independent) compared to GBLUP, likely due to redundancy between these two layers. To address this, we used the GTCBLUPi model, which addresses redundancy and provided the best model fit to our data.

### Trait phenotype predictions

The highest trait phenotype prediction accuracies were observed for models that combined genomic and transcriptomic information, for both the miRNA and mRNA data. Prediction accuracies from models incorporating both genomic and transcriptomic information were comparable to each other. Comparisons between GBLUP and two-variable models revealed significant differences in prediction accuracies. Ehsani et al. [38], Takagi et al. [41], and Perez et al. [11] also found higher prediction accuracies for mouse phenotypes when using combined data compared to GBLUP. According to these authors, the increase in accuracy by incorporating transcriptomic data in addition to genomic data in a model compared to GBLUP was highly trait dependent. Guo et al. [9] suggested that high prediction accuracy results from a high number of SNPs and transcripts, whereas our study included only a limited number of SNPs and transcript abundances. While the SNP database used in our study is smaller compared to others, we focused on highly informative transcripts, whereas other studies utilized genome-wide transcript data. Ehsani et al. [38] found that using highly expressed genes from the target tissue led to similar or even higher prediction accuracies compared to using the full set of gene expressions.

Genomic prediction is based on capturing genetic similarity between individuals using genomic relationship matrices, which enable accurate prediction [2]. Consequently, trait predictions using GBLUP were less accurate if unrelated animals were used. However, the integration of transcriptomic data holds promising potential to enhance the predictive accuracy of complex traits, remaining reliable even when using unrelated individuals.

### Biological considerations and mechanisms

The dataset used consists of a subset of a quail F2 cross experiment [20]. Principal component analysis showed no grouping or stratification and the standardized study design during rearing and phenotypic phase minimized environmental noise. The test days were considered as fixed effects in the models and in the pre-correction of the transcript abundances. Despite this, individual differences in miRNA and mRNA abundances were observed and models that incorporate transcriptomic data proved to be valuable for trait predictions. Genetic differences lead to variations in miRNA and mRNA and influence transcript abundances [17]. While SNPs provide insight into genetic variation, they only capture part of the expressed variability in phenotypes. Specifically, the 4k SNPs used in this study offer a limited representation of genome-wide genetic variation. miRNA and mRNA expression also reflect post-transcriptional regulation, including miRNA-mediated modulation, alternative splicing [42, 43], and epigenetic factors, such as DNA methylation and histone modifications, mirroring environmental and biological processes [44, 45]. Metabolic mechanisms, hormone levels, and FI may also influence gene regulation and gene expression [46–48]. Fluctuations in nutrient supply, such as P, could regulate absorption and gene expression. For instance, Vigors et al. [49] reported differences in intestinal gene expression in pigs with varying FI. The gut microbiome of individuals, which differs even under similar conditions, also influences miRNA and mRNA expression [17]. The inclusion of transcriptomic data has the potential to better capture biological mechanisms underlying trait variation, which in turn allows for more accurate prediction of trait phenotypes.

An important aspect of using transcriptomic data is sampling of both transcripts and phenotypes at the same time, as done in our dataset. Temporal alignment probably helps to capture close relationships, as gene expressions are often dynamic and time-dependent. Perez et al. [11] found that estimates of transcriptomic variance and prediction accuracy decreased as the time between phenotyping and transcript measurement increases, a finding also confirmed by Bryois et al. [50] in human transcriptomic data. Similarly, Azodi et al. [51] observed lower estimates of transcriptomic variances and limited predictive power of transcriptomic data with a long period between phenotyping and sampling for transcriptomic data collection. Therefore, recording phenotypes and transcripts at the same time allows for more accurate capture of the biological context. From a breeding perspective, transcriptomes measured at the same time as the phenotype itself do not offer additional value [52]. Nevertheless, the present study provides a proof of principle that transcriptomic information contains a substantial predictive signal for complex phenotypic traits of interest. This finding suggests the potential for the implementation of such models at earlier time points, prior to the phenotypic expression, where they could assist with predictions used for selection decisions. This approach could be valuable for traits that are expensive to measure, invasive, or only measurable later time in life. Additionally, the source of the transcriptomic data is crucial—only data that reflects the biological context of the traits, such as our ileum epithelial samples for the utilization of P, are expected to provide meaningful insights. For example, Ehsani et al. [38] observed higher prediction accuracies of physiological traits using gene expression data from the liver compared to the lung. Future work should explore the feasibility and accuracy of using transcriptomic information collected at earlier stages, potentially from accessible tissues like blood.

In the present analyses, it was assumed that the gene expression effects are normally distributed. This assumption is widely used and enables the efficient application of classical statistical methods. However, from a biological point of view, the gene expression effects might be quite heterogeneous, with a limited number of genes having a relatively large effect (as seen in Fig. 3 and Additional file 3: Figure S5). In future work, it would therefore be useful to investigate whether alternative distribution assumptions—e.g. a heavy tailed distribution—could lead to improved modeling of the data. Such models may better reflect the biological heterogeneity and thus possibly enable more precise or differentiated statements.

### GTCBLUPi model

Both Christensen et al. [19] and Perez et al. [11] addressed the fundamental challenge of redundancy between genomic and other omics layers in prediction models. The core similarity lies in their recognition that these omics are partially heritable, creating overlap between these data layers that must be accounted for to avoid double-counting genetic effects. Both approaches ultimately aim to partition phenotypic variance into components attributable to genetic effects (both direct and omics-mediated) and non-genetic effects. The key methodological difference between Christensen et al. [19] and Perez et al. [11] lies in their approach to handling this redundancy. The method of Christensen et al. [19] is inspired by the approach of Weishaar et al. [5], which employs a two-step procedure that first estimates the total effect of omics data on phenotypes and then explicitly models the genetic portion of these effects in a second step. In contrast, the GTCBLUP model of Perez et al. [11] uses a one-step approach that conditions transcripts on genotypes during modeling, effectively removing the estimated genetic component from transcripts through a smoother matrix transformation. While mathematically different, both approaches allow estimation of genetic effects while accounting for the effect of other omics data but have a different hierarchical order of modeling the different effects. Determining how similar the results of these two approaches are requires empirical comparison of the effects estimated by both models, which was beyond the scope of this paper.

Although, inspired by similar principles as Christensen et al. [19], our implementation follows the more direct conditioning approach of Perez et al. [11], while addressing some limitations in their original parameter estimation method by providing a more formal mathematical derivation and an improved parameter estimation procedure for the conditioning step. Both approaches include the parameter ${\widetilde{h}}_{t}^{2}$, which models the heritability of transcript levels on the phenotype. In the model of Christensen et al. [19] this parameter can be estimated using, e.g., Restricted Maximum Likelihood. In our implementation of the GTCBLUPi model, ${\widetilde{h}}_{t}^{2}$ is estimated by a grid search. Although it may be appealing to estimate this parameter together with all other effects, there is no apparent straightforward way to do this, given that ${\widetilde{h}}_{t}^{2}$ is an integral part of matrix ${\mathbf{W}}_{c}$, which in turn is used to compute matrix ${\mathbf{T}}_{c}$.

Trait prediction based solely on SNPs is limited to genetic similarity, which does not fully capture all biological processes that unlie observed phenotypes. This limitation becomes evident when considering the phenotypic variance explained by both genetic and transcriptomic data in a joint model. The GTCBLUPi model is suitable for incorporating intermediate omics data without modeling overlapping information between genotypes and the respective layer. Based on AIC values, it was the best-fitting model for both the miRNA and mRNA data and it explained the highest proportion of phenotypic variances and achieved the highest prediction accuracies when using the miRNA data compared to the other models. Perez et al. [11] could only observe this for some traits. In general, the GTCBLUP and GTCBLUPi models provided comparable results. Since the GTCBLUPi model more correctly adjusts the transcript levels for their estimated heritable component, the difference between the GTCBLUP and GTCBLUPi models should be particularly evident for applications where the heritabilities of the phenotypic trait and of the transcript levels are considerably different. Accurate estimation of the heritability of transcript levels may, however, require a data set with a larger number of animals than was available in our study.

The miRNA data seemed more suitable for joint modeling than the mRNA data, as using the latter resulted in lower estimates of genomic and transcriptomic variances than single modeling for some traits. In contrast, joint modeling with miRNA and genetics resulted in higher variance component estimates than single modeling for all traits except one. Other studies have also reported lower variance component estimates with joint compared to single modeling (e.g., [11, 38]). Lower variance estimates may indicate collinearity or a negative covariance between random effects, while higher variance estimates suggest a positive covariance between the random effects. Since the GTCBLUPi model addresses overlapping data, the observation that using a low heritability of transcript-level effects in GTCBLUPi was optimal (Table 2) may indicate limitations due to the size of our data. In particular, mRNA is actually influenced by many genetic factors that may not be fully captured by the limited number of SNPs. Noise in transcript data and unaccounted factors may also introduce errors. A study with more animals and SNPs would be valuable to verify the GTCBLUPi model results.

In summary, the GTCBLUPi model effectively integrates omics data, minimizing redundancy and providing the best model fit, although further studies with larger datasets are needed. The GTCBLUPi model could also be applied with other omics layers, such as the gut microbiota, to address the problem of overlapping information between genetics and the specific layer in a single BLUP model.

### Transcriptomic trait correlations

The traits in this study were genetically and phenotypically correlated [20, 24], with some pleiotropic QTL regions identified for the different traits [21]. Through the combination of discordantly expressed transcript abundances for PU and the high correlations between the traits, it is not surprising that a similar proportions of transcriptomic variance were found for the six evaluated efficiency traits.

To test the hypothesis of shared transcriptomic backgrounds, we estimated animal transcriptomic trait correlations. Despite the small sample size, we estimated both highly positive and highly negative correlations. In absolute terms, the estimated transcriptomic correlations were larger than genetic correlation estimates reported in Künzel et al. [24]. For example, the estimate of the transcriptomic correlation between PU and FCR was −0.68 (miRNA) and −0.61 (mRNA), while the genetic correlation estimate was −0.45. Notably, the differences in estimates between miRNA and mRNA correlations were small. When comparing traits with high biological dependency, such as TA and PU or CaU, we found substantially higher transcriptomic correlation estimates (close to one) than genetic correlation estimates, which were $0.50$ for TA and PU and $0.69$ for TA and CaU. One possible explanation for the stronger transcriptomic correlations compared to genetic correlations could lie in the functional nature of the traits. While SNPs capture genetic variation, they do not fully reflect the biological processes involved in mineral deposition, which depend on Ca and P absorption and storage [53]. In contrast, transcriptomic data reflect the functional activity of genes, particularly those involved in the absorption and regulation of these minerals in response to the experimental setting, i.e., lack of dietary mineral P supply. Since minerals are absorbed in the gastrointestinal tract via active transport and passive diffusion, the respective transcript data provide a more direct link between the body's homeostatic mechanisms. Thus, transcriptomic trait correlations may provide a more accurate reflection of trait relationships than genetic trait correlations, in terms of a shared functional basis for trait expression.

### Estimated effects of individual transcript abundances on phenotypes

Estimation of the effects of individual transcript abundances on phenotypes, methodology known from human studies (e.g. [54, 55]) and from a study with commercial rabbits [40], were applied here using an alternative statistical method, mirroring approaches used in microbiome trait association studies [34, 56], but with transcriptomic rather than microbial relationship matrices. The results showed that many transcripts had small and only a few had larger effects on the investigated traits. This observation aligns with the assumed polygenic origin of these traits [21]. Similarly, Jia et al. [40] identified a polygenic architecture for growth traits in meat rabbits by estimating the effect of individual transcript abundances. It is also conceivable that traits are influenced by co-expressed transcripts, suggesting coordinated transcriptomic regulation.

## Conclusion

Including both genetic and transcriptomic information in a joint model effectively explained a higher proportion of phenotypic variance of the efficiency-related traits than using the genetic information alone. The GTCBLUPi model, for which a formal derivation was presented, accounts for overlapping genetic and transcriptomic data and provided the best model fit. Transcriptomic data complemented genetic information and improved predictions of the phenotype of efficiency traits, with lower dependency on family relationships. Models that incorporated transcriptomic information outperformed the conventional GBLUP model. Additionally, estimates of transcriptomic correlations between traits were significantly higher than those of genetic correlations, with highly correlated traits influenced by the same transcripts, many with small and a few with higher transcriptomic effects.

## Supplementary Information

Additional file 1: Figure S1. Maximum log-likelihood of the GTCBLUPi model as function of the heritability of transcript-level effects (${\widetilde{h}}_{t}^{2}$) using the miRNA data. Figure S2. Maximum log-likelihood of the GTCBLUPi model as function of the heritability of transcript-level effects (${\widetilde{h}}_{t}^{2}$) using the mRNA data. Figure S3. Estimates of the proportions of variance explained by SNP genotypes (${h}^{2}$), mRNA transcript abundances (${{t}_{c}}^{2}$ / ${t}^{2}$) and the residual variance (${e}^{2}$) for the traits P utilization (PU), body weight gain (BWG), feed intake (FI), feed conversion ratio (FCR), tibia ash (TA), and Ca utilization (CaU), estimated with GBLUP, TBLUP, GTBLUP, GTCBLUP, and GTCBLUPi. For a description of the models, see Table 1.

Additional file 2: Table S1. Estimated variance components (VC) and corresponding standard errors (in parentheses) of the traits and models. Table S2. Akaike information criterion (AIC) results for each trait for different BLUP models using mRNA transcripts. Table S3. Results of the likelihood ratio tests comparing pairs of nested models. Table S4. Phenotype prediction accuracies of the BLUP models with mRNA transcripts for different traits using random cross-validation. Table S5. Phenotype prediction accuracies of the BLUP models with mRNA transcripts for different traits using family-based cross-validation.

Additional file 3: Figure S4. Estimates of differences in accuracy between the different BLUP models using mRNA transcript abundances. The average accuracies of the 500 and 45 differences are displayed as dots, the corresponding 95% confidence intervals as horizontal lines. Differences whose confidence intervals do not include zero are shown in blue. For a description of the models, see Table 1. Figure S5. Results of the effect estimation of individual transcript abundances on the phenotypes P utilization (PU), body weight gain (BWG), feed intake (FI), feed conversion ratio (FCR), tibia ash (TA), and Ca utilization (CaU). The -log10(p-values) of the mRNAs are shown. The slight red line (lower) corresponds to the significance level of p-value = 0.05 and the dark red line (upper) to the Bonferroni-corrected p-value of 0.05.

## Data Availability

The datasets used and/or analysed during the current study are available from the corresponding author on reasonable request.
