# Supplementary material for: Incorporating transcriptomic data into genomic prediction models to improve the prediction accuracy of phenotypes of efficiency traits
Source: Genet Sel Evol. 2025 Oct 23;57:59. doi: 10.1186/s12711-025-01008-7 (PMC12551188; doi:10.1186/s12711-025-01008-7)
Supplement: Supplementary file 1 — Additional file 1: Figure S1. Maximum log-likelihood of the GTCBLUPi model as function of the heritability of transcript-level effects (${\widetilde{h}}_{t}^{2}$h~t2) using the miRNA data. Figure S2. Maximum log-likelihood of the GTCBLUPi model as function of the heritability of transcript-level effects (${\widetilde{h}}_{t}^{2}$h~t2) using the mRNA data. Figure S3. Estimates of the proportions of variance explained by SNP genotypes (${h}^{2}$h2), mRNA transcript abundances (${{t}_{c}}^{2}$tc2 / ${t}^{2}$t2) and the residual variance (${e}^{2}$ [file 12711_2025_1008_MOESM1_ESM.docx]

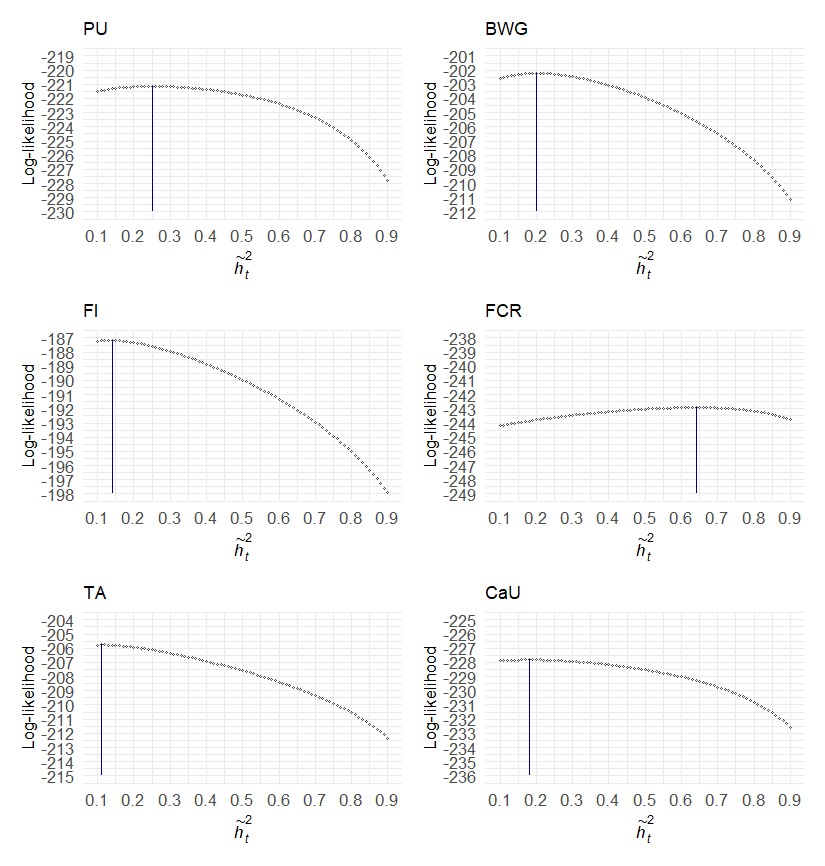


**Figure S1** Maximum log-likelihood of the GTCBLUPi model as function of the heritability of transcript-level effects ($\tilde{h}_{t}^{2}$) using the miRNA data.


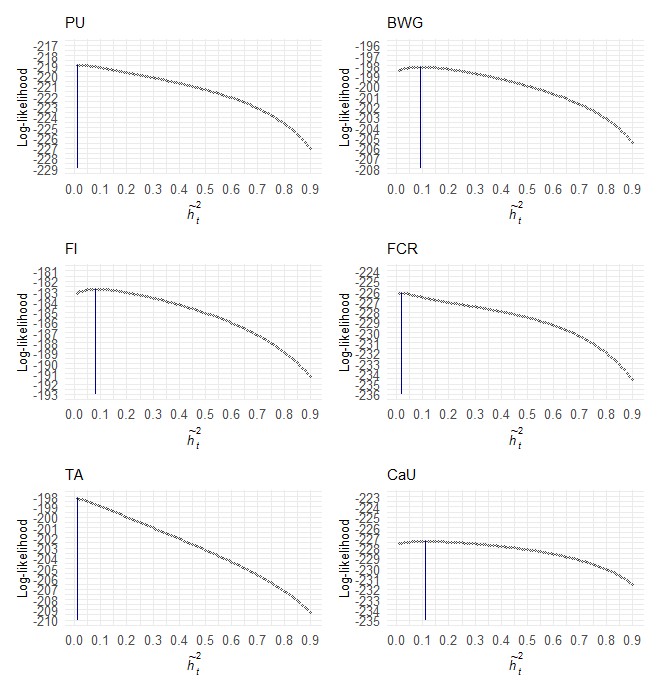


**Figure S2** Maximum log-likelihood of the GTCBLUPi model as function of the heritability of transcript-level effects ($\tilde{h}_{t}^{2}$) using the mRNA data.


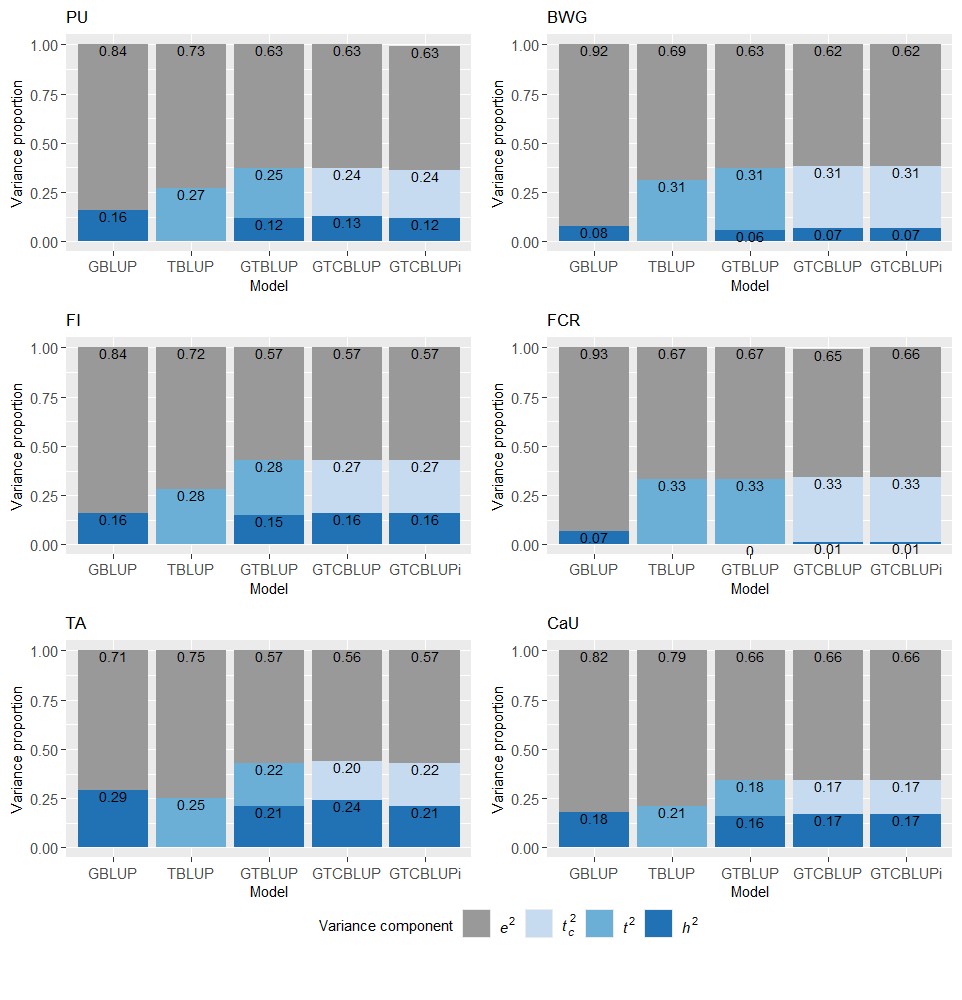


**Figure S3** Estimates of the proportions of variance explained by SNP genotypes ($h^{2}$), mRNA transcript abundances (${t_{c}}^{2}$ / $t^{2}$) and the residual variance ($e^{2}$) for the traits P utilization (PU), body weight gain (BWG), feed intake (FI), feed conversion ratio (FCR), tibia ash (TA), and Ca utilization (CaU), estimated with GBLUP, TBLUP, GTBLUP, GTCBLUP, and GTCBLUPi. For a description of the models, see Table 1.
