# Supplementary material for: Incorporating transcriptomic data into genomic prediction models to improve the prediction accuracy of phenotypes of efficiency traits
Source: Genet Sel Evol. 2025 Oct 23;57:59. doi: 10.1186/s12711-025-01008-7 (PMC12551188; doi:10.1186/s12711-025-01008-7)
Supplement: Supplementary file 2 — Additional file 2: Table S1. Estimated variance components (VC) and corresponding standard errors (in parentheses) of the traits and models. Table S2. Akaike information criterion (AIC) results for each trait for different BLUP models using mRNA transcripts. Table S3. Results of the likelihood ratio tests comparing pairs of nested models. Table S4. Phenotype prediction accuracies of the BLUP models with mRNA transcripts for different traits using random cross-validation. Table S5. Phenotype prediction accuracies of the BLUP models with mRNA transcripts for different traits using family-based cross-validation. [file 12711_2025_1008_MOESM2_ESM.docx]

**Table S1** **Estimated variance components (VC) and corresponding standard errors (in parentheses) of the traits and models**

|  |  |  | | Models | | | | | | | | | | | | | | | | | | | | | |
| --- | --- | --- | --- | --- | --- | --- | --- | --- | --- | --- | --- | --- | --- | --- | --- | --- | --- | --- | --- | --- | --- | --- | --- | --- | --- |
|  |  | only SNPs | | only miRNAs | | SNPs + miRNAs | | | | | |  | only mRNAs | | SNPs + mRNAs | | | | | | | | | | |
| Trait^a^ | VC | GBLUP | | TBLUP | | GTBLUP | | GTCBLUP | | GTCBLUPi | |  | TBLUP | | GTBLUP | | | GTCBLUP | | | GTCBLUPi | | | |  |
| PU | $Var(\mathbf{g})$ | 0.155 | (0.060) |  |  | 0.160 | (0.058) | 0.164 | (0.057) | 0.168 | (0.057) |  |  |  | 0.123 | (0.053) | 0.136 | | (0.053) | 0.126 | | (0.052) |  |  |  |
|  | $Var(\mathbf{t})$ |  |  | 0.603 | (0.206) | 0.579 | (0.200) |  |  |  |  |  | 0.279 | (0.087) | 0.255 | (0.084) |  | |  |  | |  |  |  |  |
|  | $Var(\mathbf{t}_{c})$ |  |  |  |  |  |  | 0.608 | (0.205) | 0.651 | (0.215) |  |  |  |  |  | 0.244 | | (0.081) | 0.252 | | (0.083) |  |  |  |
|  | $Var(\mathbf{e})$ | 0.816 | (0.066) | 0.794 | (0.057) | 0.640 | (0.058) | 0.630 | (0.058) | 0.618 | (0.058) |  | 0.772 | (0.055) | 0.659 | (0.058) | 0.651 | | (0.057) | 0.657 | | (0.057) |  |  |  |
| BWG | $Var(\mathbf{g})$ | 0.069 | (0.043) |  |  | 0.096 | (0.045) | 0.095 | (0.044) | 0.093 | (0.041) |  |  |  | 0.061 | (0.040) | 0.067 | | (0.039) | 0.072 | | (0.039) |  |  |  |
|  | $Var(\mathbf{t})$ |  |  | 0.763 | (0.238) | 0.838 | (0.255) |  |  |  |  |  | 0.304 | (0.089) | 0.309 | (0.091 |  | |  |  | |  |  |  |  |
|  | $Var(\mathbf{t}_{c})$ |  |  |  |  |  |  | 0.851 | (0.258) | 0.857 | (0.258) |  |  |  |  |  | 0.305 | | (0.090) | 0.304 | | (0.089) |  |  |  |
|  | $Var(\mathbf{e})$ | 0.844 | (0.064) | 0.693 | (0.050) | 0.598 | (0.053) | 0.593 | (0.053) | 0.588 | (0.052) |  | 0.679 | (0.048) | 0.622 | (0.053) | 0.616 | | (0.052) | 0.611 | | (0.052) |  |  |  |
| FI | $Var(\mathbf{g})$ | 0.144 | (0.058) |  |  | 0.160 | (0.054) | 0.156 | (0.052) | 0.156 | (0.052) |  |  |  | 0.142 | (0.054) | 0.147 | | (0.053) | 0.147 | | (0.053) |  |  |  |
|  | $Var(\mathbf{t})$ |  |  | 0.846 | (0.254) | 0.906 | (0.264) |  |  |  |  |  | 0.252 | (0.075) | 0.260 | (0.077) |  | |  |  | |  |  |  |  |
|  | $Var(\mathbf{t}_{c})$ |  |  |  |  |  |  | 0.906 | (0.262) | 0.896 | (0.259) |  |  |  |  |  | 0.250 | | (0.074) | 0.249 | | (0.074) |  |  |  |
|  | $Var(\mathbf{e})$ | 0.737 | (0.061) | 0.659 | (0.048) | 0.505 | (0.049) | 0.501 | (0.048) | 0.500 | (0.048) |  | 0.664 | (0.047) | 0.534 | (0.050) | 0.529 | | (0.050) | 0.528 | | (0.050) |  |  |  |
| FCR | $Var(\mathbf{g})$ | 0.064 | (0.050) |  |  | 0.086 | (0.057) | 0.088 | (0.057) | 0.099 | (0.057) |  |  |  | 0.000 | (0.038) | 0.016 | | (0.039) | 0.011 | | (0.038) |  |  |  |
|  | $Var(\mathbf{t})$ |  |  | 0.136 | (0.081) | 0.157 | (0.088) |  |  |  |  |  | 0.365 | (0.107) | 0.365 | (0.107) |  | |  |  | |  |  |  |  |
|  | $Var(\mathbf{t}_{c})$ |  |  |  |  |  |  | 0.164 | (0.091) | 0.191 | (0.097) |  |  |  |  |  | 0.373 | | (0.109) | 0.372 | | (0.108) |  |  |  |
|  | $Var(\mathbf{e})$ | 0.921 | (0.072) | 0.915 | (0.063) | 0.834 | (0.071) | 0.830 | (0.071) | 0.808 | (0.071) |  | 0.750 | (0.054) | 0.749 | (0.063) | 0.733 | | (0.062) | 0.737 | | (0.062) |  |  |  |
| TA | $Var(\mathbf{g})$ | 0.271 | (0.083) |  |  | 0.275 | (0.078) | 0.271 | (0.077) | 0.270 | (0.076) |  |  |  | 0.207 | (0.069) | 0.229 | | (0.071) | 0.208 | | (0.069) |  |  |  |
|  | $Var(\mathbf{t})$ |  |  | 0.458 | (0.175) | 0.514 | (0.180) |  |  |  |  |  | 0.242 | (0.076) | 0.216 | (0.072) |  | |  |  | |  |  |  |  |
|  | $Var(\mathbf{t}_{c})$ |  |  |  |  |  |  | 0.515 | (0.176) | 0.518 | (0.177) |  |  |  |  |  | 0.195 | | (0.066) | 0.212 | | (0.070) |  |  |  |
|  | $Var(\mathbf{e})$ | 0.678 | (0.063) | 0.784 | (0.056) | 0.530 | (0.056) | 0.526 | (0.055) | 0.526 | (0.055) |  | 0.726 | (0.051) | 0.552 | (0.054) | 0.543 | | (0.055) | 0.551 | | (0.054) |  |  |  |
| CaU | $Var(\mathbf{g})$ | 0.177 | (0.064) |  |  | 0.168 | (0.061) | 0.177 | (0.061) | 0.181 | (0.061) |  |  |  | 0.164 | (0.062) | 0.174 | | (0.061) | 0.176 | | (0.061) |  |  |  |
|  | $Var(\mathbf{t})$ |  |  | 0.479 | (0.180) | 0.403 | (0.161) |  |  |  |  |  | 0.224 | (0.080) | 0.183 | (0.072) |  | |  |  | |  |  |  |  |
|  | $Var(\mathbf{t}_{c})$ |  |  |  |  |  |  | 0.430 | (0.167) | 0.445 | (0.170) |  |  |  |  |  | 0.178 | | (0.069) | 0.178 | | (0.069) |  |  |  |
|  | $Var(\mathbf{e})$ | 0.793 | (0.065) | 0.833 | (0.059) | 0.677 | (0.061) | 0.664 | (0.060) | 0.658 | (0.060) |  | 0.832 | (0.059) | 0.684 | (0.061) | 0.675 | | (0.061) | 0.673 | | (0.061) |  |  |  |
| ^a^ PU: P utilization, BWG: Body weight gain, FI: Feed intake, FCR: Feed conversion ratio, TA: Tibia ash, CaU: Ca utilization.  VC: Variance component. For a description of the models, see Table 1. | | | | | | | | | | | | | | | | | | | | | | | |  |  |

**Table S2 Akaike information criterion (AIC) results for each trait for different BLUP models using mRNA transcripts**

| Trait^a^ | GBLUP | TBLUP | GTBLUP | GTCBLUP | GTCBLUPi |
| --- | --- | --- | --- | --- | --- |
| PU | 482.61^†^ | 457.87 | 443.87 | 444.14 | 443.84^‡^ |
| BWG | 466.40^†^ | 406.08 | 403.29 | 402.56 | 402.22^‡^ |
| FI | 436.49^†^ | 389.26 | 372.65 | 371.58 | 371.57^‡^ |
| FCR | 503.10^†^ | 456.64^‡^ | 458.64 | 458.34 | 458.31 |
| TA | 445.38^†^ | 426.06 | 402.39^‡^ | 404.72 | 402.40 |
| CaU | 478.57 | 482.20^†^ | 461.27 | 460.74 | 460.72^‡^ |
| ^a^ PU: P utilization, BWG: Body weight gain, FI: Feed intake, FCR: Feed conversion ratio, TA: Tibia ash, CaU: Ca utilization; **^†^** indicates the highest AIC value; **^‡^** indicates the lowest AIC value. For a description of the models, see Table 1. | | | | | |

**Table S3 Results of the likelihood ratio tests comparing pairs of nested models**^a^

| RNA data^b^ | Model comparison | | Trait^c^ | | | | | |
| --- | --- | --- | --- | --- | --- | --- | --- | --- |
|  |  |  | PU | BWG | FI | FCR | TA | CaU |
| miRNA | GBLUP | – GTBLUP | < 0.001 | < 0.001 | < 0.001 | 0.001 | < 0.001 | < 0.001 |
|  | GBLUP | – GTCBLUP | < 0.001 | < 0.001 | < 0.001 | 0.001 | < 0.001 | < 0.001 |
|  | GBLUP | – GTCBLUPi | < 0.001 | < 0.001 | < 0.001 | < 0.001 | < 0.001 | < 0.001 |
|  | TBLUP | – GTBLUP | < 0.001 | 0.001 | < 0.001 | 0.041 | < 0.001 | < 0.001 |
|  | TBLUP | – GTCBLUP | < 0.001 | < 0.001 | < 0.001 | 0.030 | < 0.001 | < 0.001 |
|  | TBLUP | – GTCBLUPi | < 0.001 | < 0.001 | < 0.001 | 0.004 | < 0.001 | < 0.001 |
| mRNA | GBLUP | – GTBLUP | < 0.001 | < 0.001 | < 0.001 | < 0.001 | < 0.001 | < 0.001 |
|  | GBLUP | – GTCBLUP | < 0.001 | < 0.001 | < 0.001 | < 0.001 | < 0.001 | < 0.001 |
|  | GBLUP | – GTCBLUPi | < 0.001 | < 0.001 | < 0.001 | < 0.001 | < 0.001 | < 0.001 |
|  | TBLUP | – GTBLUP | < 0.001 | 0.014 | < 0.001 | 0.500 | < 0.001 | < 0.001 |
|  | TBLUP | – GTCBLUP | < 0.001 | 0.009 | < 0.001 | 0.292 | < 0.001 | < 0.001 |
|  | TBLUP | – GTCBLUPi | < 0.001 | 0.008 | < 0.001 | 0.284 | < 0.001 | < 0.001 |
| ^a^ The models with a single explanatory variable (GBLUP and TBLUP) and the models with two explanatory variables (GTBLUP, GTCBLUP, and GTCBLUPi). ^b^ RNA data used to build matrix $T$ and $T_{c}$; ^c^ PU: P utilization, BWG: Body weight gain, FI: Feed intake, FCR: Feed conversion ratio, TA: Tibia ash, CaU: Ca utilization. For a description of the models, see Table 1. | | | | | | | | |

**Table S4 Phenotype prediction accuracies of the BLUP models with mRNA transcripts for different traits using random cross-validation**

|  | GBLUP | | TBLUP | | GTBLUP | | GTCBLUP | | GTCBLUPi | |
| --- | --- | --- | --- | --- | --- | --- | --- | --- | --- | --- |
| Trait^a^ | Accuracy^b^ | 95% CI^c^ | Accuracy | 95% CI | Accuracy | 95% CI | Accuracy | 95% CI | Accuracy | 95% CI |
| PU | 0.24 | 0.09:0.39 | 0.35 | 0.17:0.51 | 0.39 | 0.23:0.54 | 0.39 | 0.23:0.54 | 0.39 | 0.23:0.54 |
| BWG | 0.15 | -0.04:0.32 | 0.41 | 0.23:0.57 | 0.42 | 0.24:0.57 | 0.42 | 0.25:0.58 | 0.42 | 0.25:0.58 |
| FI | 0.23 | 0.06:0.40 | 0.40 | 0.22:0.56 | 0.46 | 0.29:0.60 | 0.46 | 0.29:0.60 | 0.46 | 0.29:0.60 |
| FCR | 0.11 | -0.05:0.26 | 0.36 | 0.19:0.52 | 0.36 | 0.19:0.51 | 0.36 | 0.19:0.51 | 0.36 | 0.19:0.51 |
| TA | 0.30 | 0.14:0.46 | 0.36 | 0.19:0.52 | 0.43 | 0.27:0.58 | 0.43 | 0.26:0.58 | 0.43 | 0.27:0.58 |
| CaU | 0.27 | 0.13:0.41 | 0.28 | 0.12:0.43 | 0.36 | 0.22:0.49 | 0.36 | 0.22:0.49 | 0.36 | 0.22:0.49 |
| ^a^ PU: P utilization, BWG: Body weight gain, FI: Feed intake, FCR: Feed conversion ratio, TA: Tibia ash, CaU: Ca utilization. ^b^ Mean accuracies. ^c^ 95% confidence intervals. For a description of the models, see Table 1. | | | | | | | | | | |

**Table S5** **Phenotype prediction accuracies of the BLUP models with mRNA transcripts for different traits using family-based cross-validation**

|  | GBLUP | | TBLUP | | GTBLUP | | GTCBLUP | | GTCBLUPi | |
| --- | --- | --- | --- | --- | --- | --- | --- | --- | --- | --- |
| Trait^a^ | Accuracy^b^ | 95% CI^c^ | Accuracy | 95% CI | Accuracy | 95% CI | Accuracy | 95% CI | Accuracy | 95% CI |
| PU | 0.00 | -0.18:0.18 | 0.29 | 0.15:0.42 | 0.30 | 0.17:0.42 | 0.30 | 0.17:0.42 | 0.30 | 0.17:0.42 |
| BWG | 0.08 | -0.10:0.26 | 0.39 | 0.24:0.53 | 0.40 | 0.26:0.53 | 0.40 | 0.25:0.53 | 0.40 | 0.25:0.53 |
| FI | 0.13 | -0.05:0.31 | 0.37 | 0.22:0.50 | 0.40 | 0.29:0.51 | 0.41 | 0.28:0.52 | 0.41 | 0.28:0.52 |
| FCR | 0.01 | -0.15:0.16 | 0.34 | 0.11:0.54 | 0.33 | 0.11:0.52 | 0.33 | 0.12:0.52 | 0.33 | 0.12:0.52 |
| TA | 0.17 | -0.07:0.40 | 0.30 | 0.11:0.47 | 0.35 | 0.17:0.51 | 0.34 | 0.17:0.49 | 0.35 | 0.17:0.51 |
| CaU | 0.02 | -0.21:0.24 | 0.20 | 0.02:0.38 | 0.22 | 0.00:0.42 | 0.23 | 0.02:0.42 | 0.23 | 0.02:0.42 |
| ^a^ PU: P utilization, BWG: Body weight gain, FI: Feed intake, FCR: Feed conversion ratio, TA: Tibia ash, CaU: Ca utilization. ^b^ Mean accuracies. ^c^ 95% confidence intervals. For a description of the models, see Table 1. | | | | | | | | | | |
