# Supplementary material for: Incorporating transcriptomic data into genomic prediction models to improve the prediction accuracy of phenotypes of efficiency traits
Source: Genet Sel Evol. 2025 Oct 23;57:59. doi: 10.1186/s12711-025-01008-7 (PMC12551188; doi:10.1186/s12711-025-01008-7)
Supplement: Supplementary file 3 — Additional file 3: Figure S4. Estimates of differences in accuracy between the different BLUP models using mRNA transcript abundances. The average accuracies of the 500 and 45 differences are displayed as dots, the corresponding 95% confidence intervals as horizontal lines. Differences whose confidence intervals do not include zero are shown in blue. For a description of the models, see Table 1. Figure S5. Results of the effect estimation of individual transcript abundances on the phenotypes P utilization (PU), body weight gain (BWG), feed intake (FI), feed conversion ratio (FCR), tibia ash (TA), and Ca utilization (CaU). The -log10(p-values) of the mRNAs are shown. The slight red line (lower) corresponds to the significance level of p-value = 0.05 and the dark red line (upper) to the Bonferroni-corrected p-value of 0.05. [file 12711_2025_1008_MOESM3_ESM.docx]

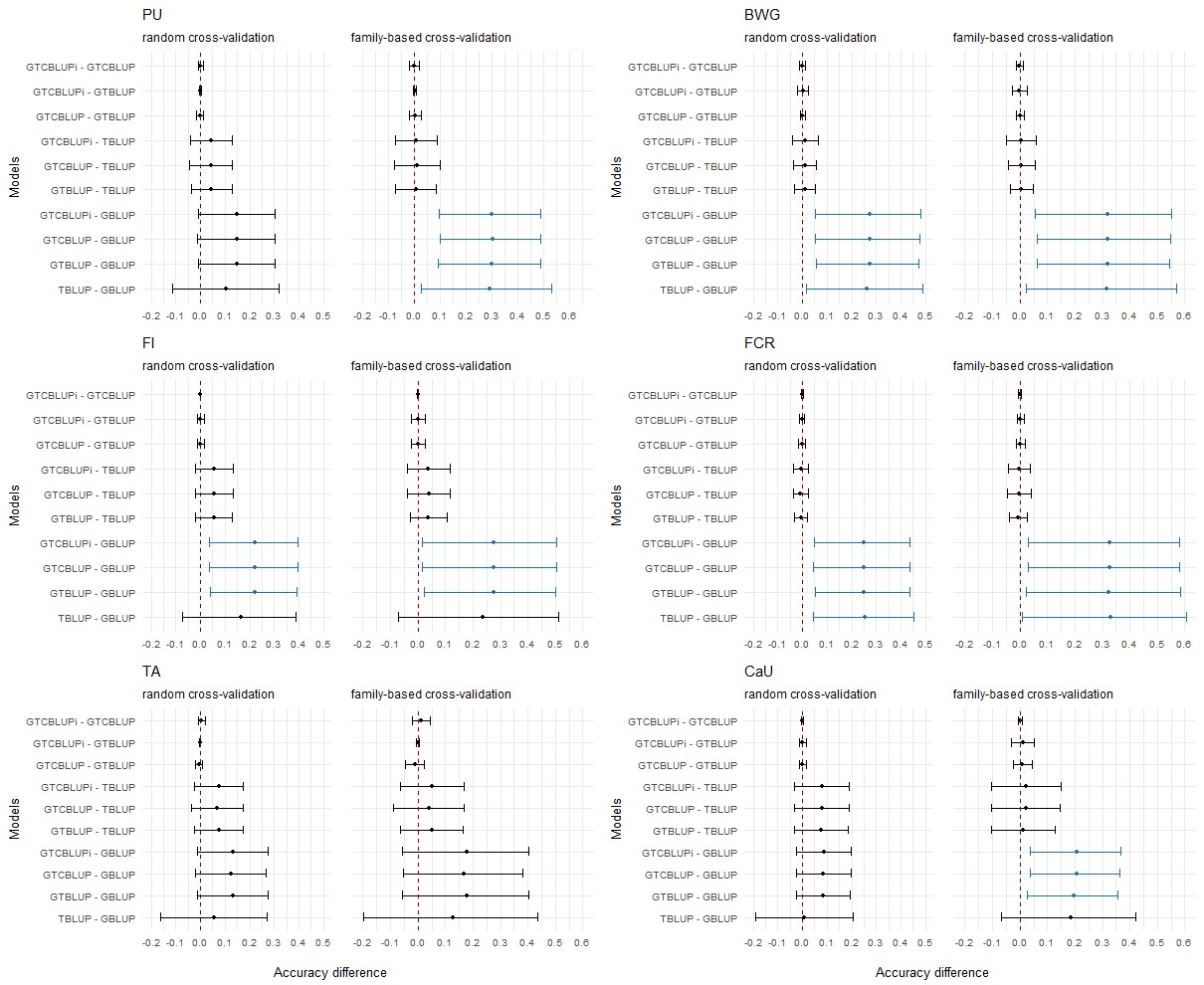


**Figure S4** Estimates of differences in accuracy between the different BLUP models using mRNA transcript abundances. The average accuracies of the 500 and 45 differences are displayed as dots, the corresponding 95% confidence intervals as horizontal lines. Differences whose confidence intervals do not include zero are shown in blue. For a description of the models, see Table 1.


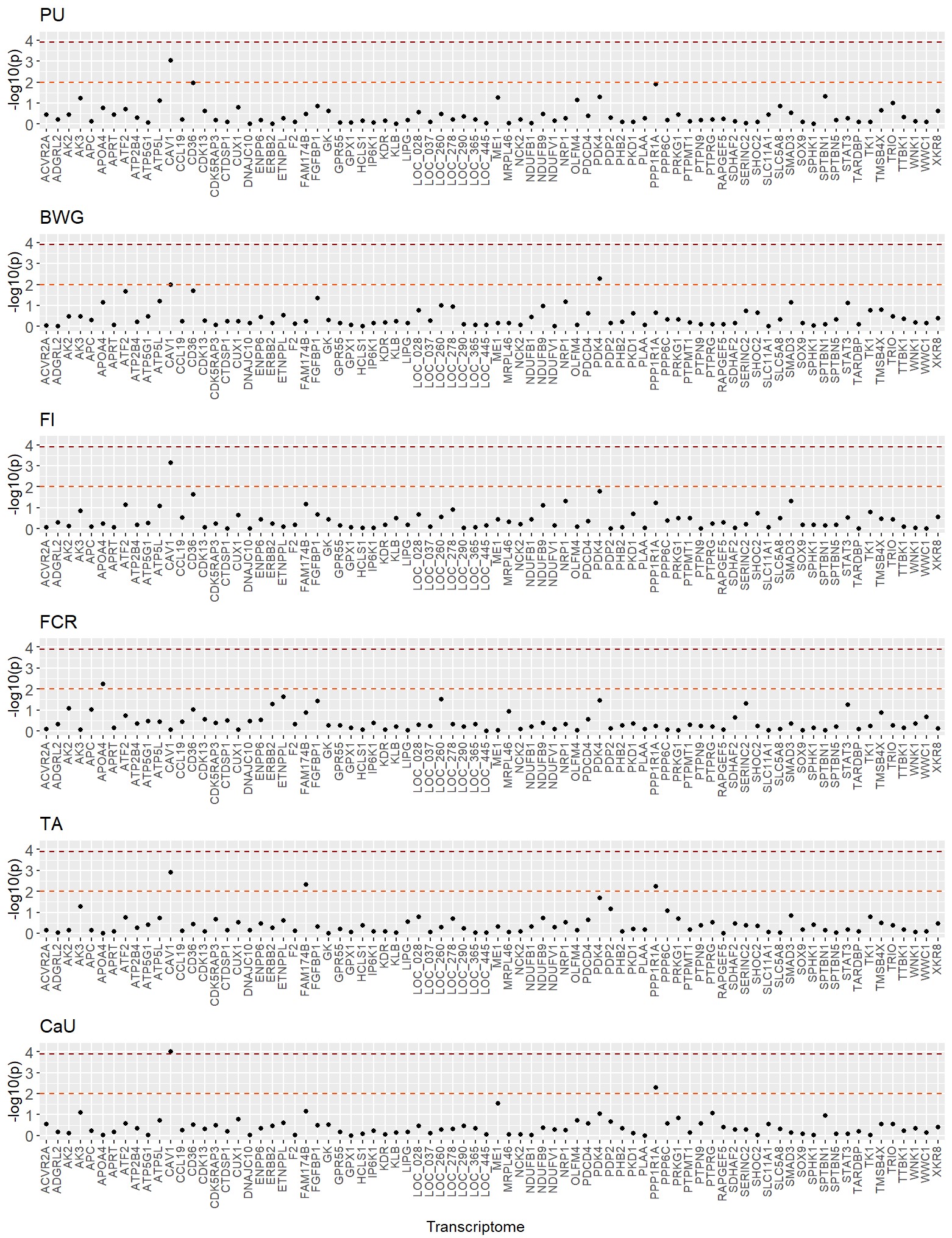


**Figure S5** Results of the effect estimation of individual transcript abundances on the phenotypes P utilization (PU), body weight gain (BWG), feed intake (FI), feed conversion ratio (FCR), tibia ash (TA), and Ca utilization (CaU). The -log10(p-values) of the mRNAs are shown. The slight red line (lower) corresponds to the significance level of p-value = 0.05 and the dark red line (upper) to the Bonferroni-corrected p-value of 0.05.
